# A multitargeting nanoparticle-based vaccine for enhanced anticancer immunotherapy in HER2-Positive breast cancer

**DOI:** 10.1016/j.mtbio.2026.103524

**Published:** 2026-08-03

**Authors:** Sen Liu, Yiqiang Zhu, Tao Chen, Xiaoqing Liu, Jintao Lai, Meilin Hu, Yaoming Liu, Haiyue Rao, Bin Zhang, Shiqi Xiao, Haojie Peng, Taizhen Liang, Lixiang Xie, Guochang Qiu, Shan Li, Xiancai Ma

**Affiliations:** a School of Biology and Biological Engineering, South China University of Technology, Guangzhou, 510006, China; b Institute of Human Virology, Zhongshan School of Medicine, Sun Yat-sen University, Guangzhou, 510080, China; c Guangzhou National Laboratory, Guangzhou International Bio-Island, Guangzhou, 510005, China; d State Key Laboratory of Respiratory Disease, National Clinical Research Center for Respiratory Disease, Guangzhou Institute of Respiratory Health, The First Affiliated Hospital of Guangzhou Medical University, Guangzhou, 510120, China

**Keywords:** Cancer immunotherapies, Nanoparticle vaccines, Immune checkpoint blockade therapies, HER2-Positive breast cancers, Ferritin

## Abstract

Tumor vaccines represent promising strategies for cancer immunotherapy. However, their application remains restricted in HER2-positive breast cancer due to poor immunogenicity and inefficient antigen presentation. Herein, a multitargeting HER2 nanoparticle vaccine, HER2_aHPF, is developed by co-displaying the HER2 extracellular domain and AE37 T-cell epitope on a *Helicobacter pylori* ferritin nanoparticle via SpyTag/SpyCatcher conjugation. The nanoparticle vaccine exhibits structural homogeneity, enhanced lymphatic drainage, and high stability during freeze-thaw cycles and long-term storage, with retained immunogenicity after repeated freeze-thaw treatment. WH peptide-mediated DC targeting, in combination with the ferritin scaffold, enhances antigen uptake and DC maturation. In BALB/c mice, CpG and fullerenol dual-adjuvanted HER2_aHPF elicits robust HER2-specific humoral and cellular immunity, characterized by high-titer antibodies, polyfunctional CD4^+^ and CD8^+^ T cells, and expanded effector-memory T cells. Prophylactic and therapeutic vaccination markedly inhibits primary tumor growth and lung metastasis in mouse and human HER2-positive 4T1 models. Mechanistically, HER2_aHPF reduces myeloid-derived suppressor cells and regulatory T cells, increases cytotoxic T-cell infiltration, and maintains a favorable *in vivo* safety profile. Combination with anti-PD-1 and anti-CD47 antibodies further enhances T-cell activity and tumor control, demonstrating strong synergy between active vaccination and dual immune checkpoint blockade. These findings establish HER2_aHPF as a versatile nanoparticle vaccine platform with clinical translation potential for HER2-positive cancer immunotherapy.

## Highlights

• A multitargeting HER2_aHPF nanoparticle vaccine is rationally designed.
• HER2_aHPF elicits robust HER2-specific humoral and cellular immune responses.
• HER2_aHPF demonstrates both prophylactic and therapeutic efficacy.
• HER2_aHPF synergizes with ICB to enhance anti-tumor activity.

## Graphical abstract

Shan Li, Xiancai Ma, and their colleagues develop a multitargeting HER2 nanoparticle vaccine that induces potent cellular and humoral immune responses. Both prophylactic and therapeutic administrations of HER2 nanoparticle vaccines inhibit tumor growth and synergize with immune checkpoint blockade therapies to enhance tumor suppression, highlighting translational potentials of nanoparticle vaccines against cancers.

**Figure:**
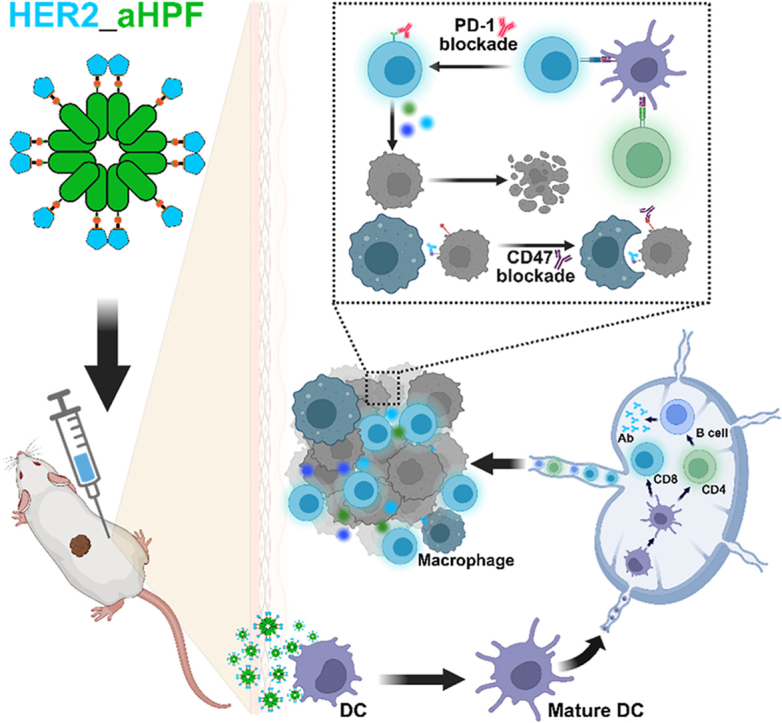

## 1 Introduction

Breast cancer persists as the most frequently diagnosed malignancy among women worldwide and remains a leading cause of cancer-related mortality [1]. Among its molecular subtypes, human epidermal growth factor receptor 2 (HER2)-positive breast cancer represents a particularly aggressive variant, accounting for approximately 15-20% of cases and characterized by rapid disease progression and unfavorable prognosis [2]. This subtype is defined by HER2 gene amplification and consequent protein overexpression, which drives tumorigenesis through constitutive activation of downstream signaling pathways that promote uncontrolled cellular proliferation and invasion. In addition to breast cancer, aberrant HER2 amplification or overexpression has also been observed in various other tumor types, including gastric cancer, biliary tract cancer, bladder cancer, colorectal cancer, and non-small-cell lung cancer (NSCLC) [3,4]. The development of HER2-targeted therapies, most notably the monoclonal antibodies (mAbs) trastuzumab, pertuzumab, and margetuximab, has substantially improved patient survival outcomes [[5], [6], [7]]. Nevertheless, these passive immunization approaches face significant limitations, including the emergence of both primary and acquired resistance, high recurrence rates, and persistent risk of distant metastasis to critical organs such as the lungs, liver, and brain [[8], [9], [10], [11]]. Mechanisms of resistance encompass intratumoral HER2 heterogeneity, truncated HER2 isoforms, activating mutations or loss of PTEN leading to reactivation of PI3K/AKT signaling, compensatory upregulation of parallel receptor tyrosine kinases, and immune-evasion mechanisms that impair antibody-dependent cellular cytotoxicity (ADCC). To address these gaps, modern therapeutic strategies, including dual HER2 blockade, antibody-drug conjugates (ADCs), and rational combinations with pathway inhibitors, have effectively targeted these vulnerabilities [[12], [13], [14]]. Importantly, active immunotherapies, including cancer vaccines, bispecific T-cell engagers, and adoptive cell therapies, hold promise for establishing durable systemic immunity, enabling the eradication of micrometastases and preventing relapse [[15], [16], [17]].

Active cancer immunotherapy, particularly through therapeutic vaccination, represents a paradigm-shifting approach that leverages the patient's immune system to specifically recognize and eliminate malignant cells [18,19]. In HER2-positive breast cancer, therapeutic vaccines have emerged as a promising immunotherapeutic strategy. Multiple vaccine platforms incorporating peptide or subunit antigens have demonstrated both preclinical efficacy and early clinical activity. HER2 protein-derived synthetic peptide vaccines, such as E75, GP2, and AE37, have been found to boost CD4^+^ T helper and CD8^+^ cytotoxic T lymphocyte (CTL) responses in clinical trials [[20], [21], [22]]. Conversely, protein subunit vaccines based on recombinant HER2 protein fragments, such as the full extracellular domain (ECD) or the intracellular domain (ICD), offer broader epitope coverage and have been shown to elicit both humoral and cellular immune responses in patients with HER2-positive breast cancer [23,24]. However, immune responses elicited by these vaccines differ in magnitude and durability, and have not consistently led to broad or definitive clinical benefits when used as monotherapy. Major challenges include suboptimal antigen presentation by dendritic cells (DCs), limited cross-presentation to T lymphocytes, insufficient generation of long-lived memory responses, and suppression of vaccine-induced immunity within the highly immunosuppressive tumor microenvironment [[25], [26], [27], [28]]. These limitations highlight the urgent need for innovative vaccine strategies that can overcome these biological barriers through advanced engineering approaches.

Recent advances in nanotechnology have opened new avenues for addressing challenges associated with conventional cancer vaccines [29]. Both organic and inorganic nanoparticle-based vaccine platforms have demonstrated transformative potential for advancing modern immunotherapy by effectively overcoming major obstacles in antigen delivery, immunological tolerance, and immune activation [30]. Their nanostructures facilitate lymphatic drainage and enable targeted antigen delivery to antigen-presenting cells (APCs) while protecting antigen integrity from degradation and ensuring high payload capacity [31]. In addition, nanoparticle vaccines can enhance MHC-I antigen presentation by escaping from endosomes and accessing the cytosol of APCs, thereby promoting CD8^+^ T-cell priming and cytotoxic immune responses [32]. Within lymph nodes, nanoparticle vaccines can be presented by follicular dendritic cells (FDCs) to B cells in intact form, enabling native conformational epitope presentation, enhancing B-cell receptor (BCR) cross-linking, consequently facilitating T follicular helper (Tfh)-B cell interactions and improving germinal center (GC) responses [33]. Moreover, these systems allow precise control over antigen orientation and surface density, and can be functionally modularized to co-deliver immunostimulatory agents or APC-targeting peptides, thereby markedly enhancing immune targeting, immunogenicity, and therapeutic outcomes [[34], [35], [36]].

Among various nanoplatforms, protein-based nanoparticles have garnered significant attention owing to their excellent biocompatibility, precise self-assembly characteristics, and well-defined structural configurations [37]. Protein nanoparticles have been extensively utilized to deliver viral antigens for combating infectious diseases [38]. Commonly used protein nanoparticles include ferritin, lumazine synthase (LS), I53-50, mi3, and dihydrolipoyl acetyltransferase (E2p) [[39], [40], [41], [42], [43]]. Vaccines based on these nanoparticles have shown significantly enhanced immunogenicity compared to their monomeric counterparts. Recently, Dingkang Liu et al. designed a ferritin-based protease-activated PSTAGylated *in situ* tumor vaccine (PPTV) for breast cancer via co-delivery of mito-disrupt peptides and manganese ions (Mn^2+^) [44]. PPTV activated both immunogenic cell death and cGAS-STING pathways and synergized with the anti–programmed death-ligand 1 (αPD-L1) therapy to achieve potent anti-tumor efficacy. The filamentous potato virus X (PVX) nanoparticle has been employed to deliver the B-cell epitope of HER2, thereby enhancing immunological stability and improving B-cell epitope presentation [45]. Another study revealed that HER2 epitopes displayed on icosahedral cowpea mosaic virus (CPMV) nanoparticles lead to enhanced APC activation and lymph node targeting [46]. Arianna Palladini et al. utilized an AP205 phage-derived nanoparticle in conjunction with the SpyTag/SpyCatcher (ST/SC) system to covalently present 183 copies of HER2-ECD [47]. Prophylactic vaccination of this nanoparticle vaccine reduced the development of HER2-positive mammary carcinoma and suppressed tumor growth. These groundbreaking achievements in infectious disease and cancer vaccinology demonstrate the versatility and potential of protein nanoparticles as a highly effective vaccine platform, providing an innovative technological approach to address the challenge of insufficient immunogenicity.

**Scheme 1:**
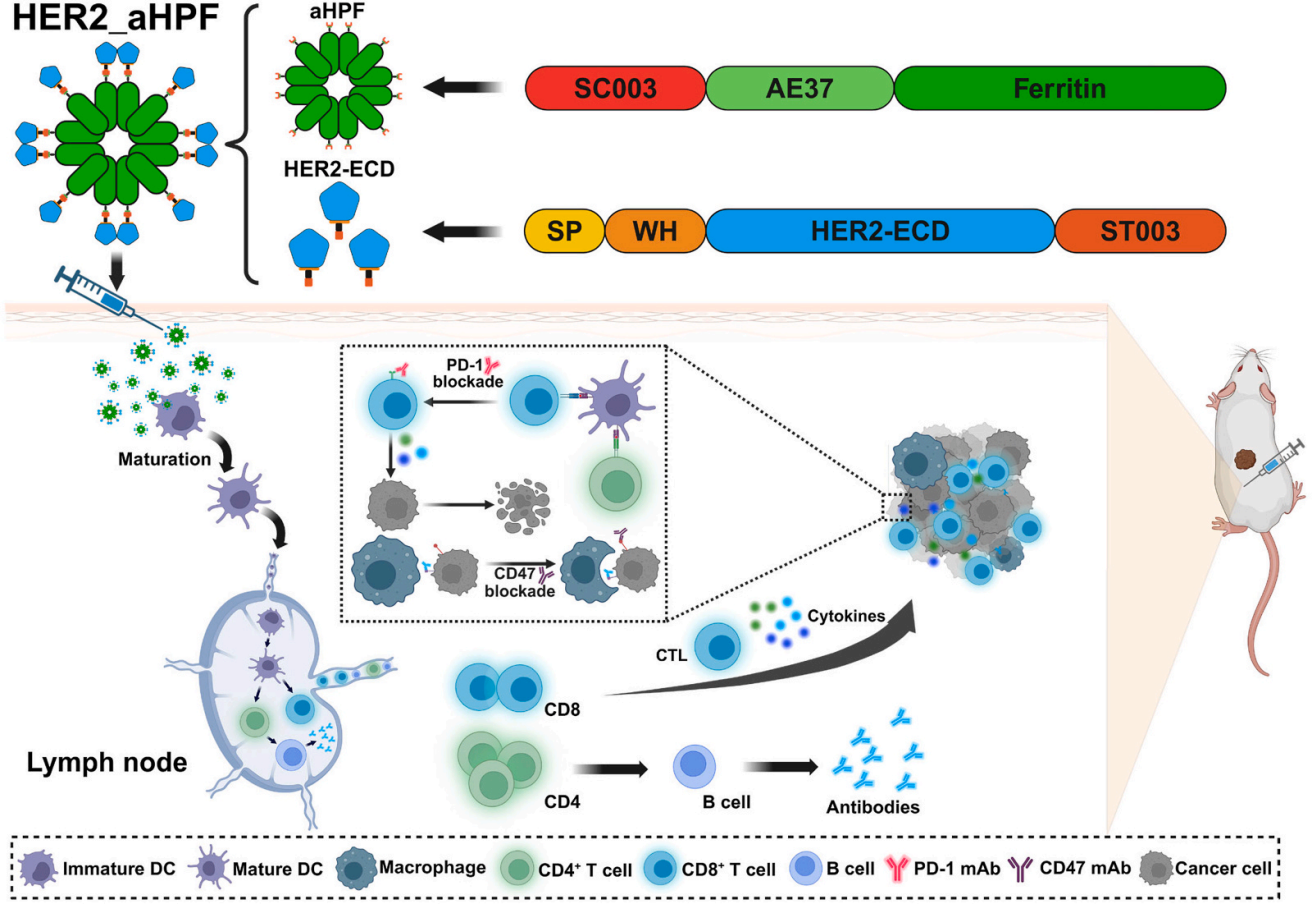
**Schematic illustration of the construction of a multitargeting HER2 nanoparticle vaccine and its underlying mechanisms of anticancer immunotherapy.***In vitro* purified AE37-conjugated *Helicobacter pylori* ferritin (HPF) and WH peptide-conjugated HER2-ECD were covalently conjugated via the ST003/SC003 system to construct the HER2_aHPF nanoparticle vaccine. This vaccine promoted DC maturation and augmented cellular and humoral immune responses, as evidenced by inducing higher percentages of CD80^+^CD86^+^ mature DCs, cytokine-secreting polyfunctional T cells, and HER2-specific antibodies when compared to the monomeric vaccine. Both prophylactic and therapeutic vaccinations of the HER2_aHPF vaccine synergized with anti-PD-1/anti-CD47 immune checkpoint blockade (ICB) therapies to effectively prevent the progression of mouse or human HER2-positive tumors.

Based on these advantages of nanoparticles, we designed and characterized a multitargeting nanoparticle vaccine that coordinately activated humoral and cellular immunity against HER2-positive breast cancer (Scheme 1). Considering that the ECD of HER2 harbors both B-cell epitopes targeted by therapeutic mAbs such as trastuzumab and pertuzumab and the T-cell epitope E75 that induces potent CTL responses, twenty-four copies of HER2-ECD antigens were covalently conjugated to the surface of *Helicobacter pylori* ferritin (HPF) via the SpyTag003/SpyCatcher003 (ST003/SC003) system [20,48,49]. Another T-cell epitope AE37 derived from HER2-ICD was expressed in-frame with HPF to potentiate HER2-specific CD4^+^ T-helper responses [22]. To facilitate antigen presentation, a WH peptide targeting Clec9a on DCs was conjugated at the N-terminal of HER2-ECD [50]. Previous studies have demonstrated that both CpG oligodeoxynucleotide and fullerenol nanoparticle (FNP) have potent immunostimulatory effects and potentiate clearance of cancer cells [51,52]. In this study, we integrated a dual-adjuvant system comprising CpG and FNP into the HER2 nanoparticle vaccine.

The integrated nanoparticle vaccine formulations augmented both cellular and humoral immune responses against mouse and human HER2 antigens relative to monomeric HER2 vaccines (Scheme 1). Mechanistically, the vaccines exhibited enhanced lymphatic drainage and lymph node retention, promoted antigen uptake and DC maturation, and elicited robust polyfunctional T-cell responses and HER2-specific antibody production. Both mHER2_aHPF and hHER2_aHPF maintained structural integrity and immunogenicity after repeated freeze-thaw cycles and prolonged storage. A comprehensive safety evaluation revealed no evidence of organ toxicity or pathological systemic inflammation. Head-to-head comparison with trastuzumab demonstrated superior tumor suppression by hHER2_aHPF vaccination. Prophylactic and therapeutic vaccination effectively suppressed primary tumor growth and pulmonary metastasis. Combination with anti-PD-1 and anti-CD47 ICB therapies further reshaped the immunosuppressive tumor microenvironment by decreasing MDSCs and Tregs while increasing tumor-infiltrating polyfunctional CD4^+^ and CD8^+^ T cells. These findings indicate that the ferritin-based nanoparticle vaccine incorporating the CpG-FNP dual-adjuvant system elicits coordinated anti-tumor immunity. Upon combination with dual ICB therapy, this vaccine overcomes key immunosuppressive barriers to achieve superior tumor control and metastasis suppression.

## 2 Materials and methods

### 2.1 Materials and reagents

*Escherichia coli* (*E. coli*) strains DH5α (KTSM101L) and BL21 (DE3) (KTSM104L) competent cells were purchased from AlpaLifeBio Technology Co., Ltd. (Shenzhen, China). CpG ODN 1826 (HY-146245) was purchased from MedChemExpress (Monmouth Junction, NJ, USA). Fullerenol (793248) was purchased from Merck KGaA (Darmstadt, Germany). SMM 293-TII Expression Medium (M293TII) and SMS 293-SUPI Expression Medium Supplement (M293-SUPI) were purchased from Sino Biological, Inc. (Beijing, China). RPMI 1640 (L210KJ) and DMEM (L110KJ) media were purchased from Shanghai Basal Media Co., Ltd. (Shanghai, China). Hyaluronidase (abs47014926) was purchased from Absin Bioscience Inc. (Shanghai, China). IL-4 (214-14-100UG) and GM-CSF (315-03-100UG) were purchased from PeproTech (Rocky Hill, NJ, USA). Anti-PD-1 (RMP1-14, BE0146) and anti-CD47 (MIAP301, BE0270) antibodies were purchased from BioXcell (Lebanon, New Hampshire, USA). D-Luciferin potassium salt (LS002) was purchased from Guangzhou Biolight Biotechnology Co., Ltd. (Guangzhou, China). Triangular shake flasks (TAB002000) were purchased from Guangzhou Jet Bio-Filtration Co., Ltd. (Guangzhou, China). YoungPAGE Precast Gels (M00653) were purchased from GenScript Biotechnology Co., Ltd. (Nanjing, China). Fluorescent dye-conjugated anti-mouse antibodies against CD3-FITC (145-2C11, 100306), CD3-AF700 (500A2, 152316), CD80-APC (16-10A1, 104713), CD86-APC-Cy7 (GL-1, 105030), CD62L-PE-Cy7 (MEL-14, 104417), CD44-BV650 (IM7, 103049), CD8α-PE (53-6.7, 100708), Gr-1-FITC (RB6-8C5, 108405), IFNγ-BV421 (XMG1.2, 505830), TNF-α-APC (MP6-XT22, 506307), CD8α-PE-Cy7 (53-6.7, 100722), CD4-AF700 (RM4-5, 100536), CD45-APC-Cy7 (30-F11, 103116), CD11b-PE-Cy7 (M1/70, 101216), CD25-APC (PC61, 102011), CD107a-BV421 (1D4B, 121617), Granzyme B-PE (QA16A02, 372207), and CD11c-PerCP-Cy5.5 (N418, 117328) for flow cytometry analysis were purchased from BioLegend (San Diego, CA, USA). Anti-mouse Foxp3-PE (R16-715, 563101) was obtained from BD Biosciences (Franklin Lakes, NJ, USA).

### 2.2 Mice and cell lines

4T1 murine breast cancer cell lines (TCM32) were obtained from the Cell Bank of the Chinese Academy of Sciences (Shanghai, China). Stable 4T1 monoclonal cells expressing murine or human HER2 were generated by lentiviral transduction of the pLVX-EF1α-HER2-P2A-puro-luc construct followed by puromycin selection. DC2.4 mouse dendritic cell lines and RAW264.7 mouse macrophage cell lines were kindly provided by Professor Jingyou Yu and Professor Qiong Zhang at Guangzhou National Laboratory, respectively. 4T1 cells and DC2.4 cells were grown in RPMI 1640 medium with 10% fetal bovine serum (FBS) (FSP500, ExCell Bio, China) and 1% penicillin-streptomycin (15140163, Gibco, USA) solution. RAW264.7 cells were cultured in DMEM medium containing 10% FBS and 1% penicillin-streptomycin. All the above-mentioned cells were maintained in a humidified environment at 37°C and 5% CO_2_. HEK293F cell lines (R79007, Gibco) were cultured in SMM 293-TII Expression medium supplemented with 1% penicillin-streptomycin, and incubated in a shaker at 37°C, 8% CO_2_, and 120 rpm. All cells used in this study were confirmed to be mycoplasma-free by PCR-based testing. Female BALB/c mice (6-8 weeks) (N000020) were obtained from GemPharmatech Co., Ltd. (Nanjing, China). All animal procedures were performed in accordance with animal ethics standards and were approved by the Animal Care and Use Committee of Guangzhou National Laboratory (Approval No. GZLAB-AUCP-2025-06-A03).

### 2.3 Plasmid construction

The genes encoding the extracellular domains (ECDs) of mouse or human HER2 proteins were synthesized by Sino Biological, Inc. The SpyTag003 (ST003) and 6×hexahistidine (6×His) tags were fused at the C-terminal of HER2-ECD, while the signal peptide (SP) (MGILPSPGMPALLSLVSLLSVLLMGCVA), WH peptide (WPRFHSSVFHTH), and 5×GGGGS linkers were sequentially integrated into the N-terminal region. The constructs were then cloned into the pcDNA3.1-Intron-IRES-GFP-WPRE vector. The SC003-AE37-HPF clone was obtained by fusing SpyCatcher003 (SC003) tag and AE37 peptide (LRMKGVGSPYVSRLLGICL) to the N-terminal of *Helicobacter pylori* ferritin (HPF), and a 6×His tag to the C-terminal of HPF. The construct was then cloned into the pET28a vector.

### 2.4 Recombinant protein expression and purification

SC003-AE37-HPF proteins were expressed by prokaryotic expression system. The constructed pET28a-SC003-AE37-HPF-6×His plasmid was transformed into *E. coli* BL21 (DE3) cells. The transformed cells were then plated on a LB agar plate containing 50 μg/mL kanamycin and incubated at 37°C overnight. A single colony was picked and inoculated into 400 mL LB medium supplemented with kanamycin, then cultured at 37°C with shaking at 220 rpm until OD600 reached 0.4. 1 mM of isopropyl β-D-1-thiogalactopyranoside (IPTG) was added to the culture to induce protein expression at 16°C for 14 h (h). After induction, bacterial cells were harvested and resuspended in a buffer consisting of 20 mM Tris-HCl and 50 mM NaCl. The bacterial cells were lysed using a low-temperature ultra-high-pressure cell disruptor, and the supernatant was collected by centrifugation. After the supernatant was passed through a 0.45 μm filter membrane, the supernatant was further incubated with TALON Superflow beads (Cytiva) for 1 h at 4°C with gentle shaking. The enriched target proteins were eluted with an imidazole-containing buffer (20 mM Tris-HCl, 50 mM NaCl, 300 mM Imidazole, pH 7.5). The eluted fractions were concentrated and buffer-exchanged with an imidazole-free buffer (20 mM Tris-HCl, 50 mM NaCl, pH 7.5).

The mouse and human recombinant WH-HER2-ST003-6×His proteins were expressed and purified from mammalian cell expression system. Briefly, the pcDNA3.1-Intron-SP-HER2-ST003-6×His-IRES-GFP-WPRE plasmid was transfected into HEK293F cells with polyethylenimine Max (PEIMAX) reagent. Following 7 days of culture, the supernatant was collected via centrifugation at 9000 rpm and 4°C for 5 min (min). The filtered supernatant was purified using Ni Sepharose Excel beads (Cytiva, USA), and the target protein was eluted with a buffer containing 20 mM Tris, 500 mM imidazole, and 50 mM NaCl, with pH adjusted to 7.5. The eluted protein solution was concentrated and buffer-exchanged with an imidazole-free buffer (20 mM Tris-HCl, 50 mM NaCl, pH 7.5).

### 2.5 Size-exclusion chromatography

WH-mHER2-ST003 or WH-hHER2-ST003 was incubated with SC003-AE37-HPF at molar ratios of 1:1, 1:2, 1:3, and 1:4 at 4°C. After incubating for 24 h, the conjugated proteins were analyzed using size-exclusion chromatography (SEC) with a Superose 6 Increase 10/300 GL column (Cytiva, USA) in 20 mM Tris, 50 mM NaCl buffer (pH 7.5). The major peaks of SC003-AE37-HPF appeared at retention volumes of 10-13 mL, while mHER2-ST003 or hHER2-ST003-conjugated nanoparticles were eluted at retention volumes of 8-10 mL. The eluates of antigen-conjugated nanoparticles were collected and subjected to centrifugal filtration to obtain proteins of higher purity using a 100 kDa ultrafiltration tube (Pall, USA). Concentrations of these nanoparticle vaccines were determined by BCA assays, while purities were confirmed by Coomassie blue staining.

### 2.6 3D rendering and charge analysis of nanoparticles

The rendered three-dimensional (3D) structures of aHPF, mHER2_aHPF, and hHER2_aHPF were predicted using AlphaFold 3. During each prediction, multiple model seeds were employed in the modeling process under the condition that an isopeptide bond forms between ST003 and SC003. Models with the highest predicted confidence scores were selected for subsequent analysis. The highest-confidence complex structures were visualized and rendered using UCSF ChimeraX software (version 1.10). Molecular surface electrostatic potentials of each nanoparticle were calculated and analyzed with the APBS Electrostatics plugin within PyMOL software (version 3.1.6.1). The calculation parameters were set as follows: the AMBER force-field was applied, and the electrostatic potential isosurface was displayed within a range of ± 5.0 kT/e (color-coded blue for positive and red for negative regions).

### 2.7 Dynamic light scattering

The hydrodynamic diameters of nanoparticle vaccines were determined by dynamic light scattering (DLS) using a Zetasizer Ultra instrument (Malvern Panalytical, UK). Briefly, the nanoparticle samples were diluted with PBS to a final concentration of 0.1 mg/mL and loaded into a quartz cuvette. After a 1-min equilibration period at 25°C, the measurements were performed 5 times with a 30-s (s) duration for each run. The diffusion coefficient was derived from the analysis of scattered light intensity fluctuations via an autocorrelation function, which was subsequently used to calculate the nanoparticle size. The zeta potential of the nanoparticles was measured by electrophoretic light scattering using the same instrument. Samples were diluted with PBS to a concentration of 0.5 mg/mL. Upon application of an electric field, the electrophoretic mobility of the charged particles was determined by analyzing the frequency shift of the scattered light. The zeta potential was then automatically calculated from the mobility by the integrated Zetasizer Ultra ZS Xplore software.

### 2.8 Negative staining

The morphology of nanoparticles was characterized by negative staining using a Talos L120C microscope (ThermoFisher, USA) operated at 120 kV. The samples (0.025 μg/μL) were applied onto glow-discharged copper grids coated with a continuous carbon film. This was performed by four successive applications of 4 μL aliquots, with excess solution blotted away after each step. The grids were then negatively stained with 2% uranyl acetate, also in three repeats. Images were acquired at a magnification of 73,000× and a defocus value of 1.5 μm. Automated particle picking and two-dimensional (2D) classification were performed using cryoSPARC. A total of 1,884, 3,705, and 7387 particles were automatically identified and analyzed for aHPF, mHER2_aHPF, and hHER2_aHPF, respectively. Particle diameters were determined from the 2D class averages.

### 2.9 Protein stability

To assess the freeze-thaw stability of mHER2-or hHER2-conjugated AE37-HPF nanoparticles (mHER2_aHPF or hHER2_aHPF), the nanoparticles were subjected to 1 to 9 cycles of freeze-thaw treatment, with temperature transitioning from −80°C to 37°C. Subsequently, the samples were centrifuged at 12,000 rpm for 10 min at 4°C. The content of soluble proteins in the supernatant was determined using a BCA protein quantification kit (Thermo Fisher, USA). For the study on storage condition stability, protein samples were assigned to different storage protocols: storage at −80°C for 2 months, −20°C for 1 month, or 4°C for 1 week. Samples were centrifuged to clear potential aggregates. The supernatant proteins were resolved via SDS-PAGE, with the target protein bands visualized. After Coomassie blue staining, protein bands were visualized and images of gels were captured. For each lane, the integrated intensity of the intact protein band was divided by the total lane intensity (intact plus degraded bands) to calculate the percentage of intact protein using ImageJ software.

### 2.10 SDS-PAGE and native PAGE analysis

Protein samples were mixed with 5×loading buffer, boiled at 100°C for 10 min, and separated on the YoungPAGE Precast Gels at 200 V for 30 min. After separation, the gels were stained with Coomassie Brilliant Blue solution for 1 h, destained in deionized water, and imaged. For Native PAGE analysis, conjugated nanoparticles were firstly mixed with native loading buffer. The samples were directly loaded without heat treatment and separated on Native PAGE Bis-Tris Gels (Thermo Fisher, USA) at 140 V for 2 h at 4°C. Gels were stained with Coomassie Brilliant Blue solution and destained until clear bands appeared. And protein band images were acquired.

### 2.11 Endotoxin content testing

Endotoxin levels were determined using a chromogenic Tachypleus amebocyte lysate (TAL) assay (Bioendo EC Endotoxin Test Kit, EC80545S, Xiamen Bioendo Technology Co., Ltd., China) according to the manufacturer's instructions. Briefly, aHPF, mHER2_aHPF, and hHER2_aHPF from three independent batches per preparation were diluted with endotoxin-free water and mixed with the provided TAL reagents. After incubation with the chromogenic substrate, absorbance was measured at 545 nm. Endotoxin concentrations were calculated by interpolation from a standard curve ranging from 0.1 to 1.0 EU/mL and expressed as endotoxin units per milliliter (EU/mL). The pharmacopoeial limit for parenteral products is 5 EU per kg of body weight per hour, as specified by the United States Pharmacopoeia (USP <85>), European Pharmacopoeia (Ph. Eur. 2.6.14), and Chinese Pharmacopoeia (ChP 1143).

### 2.12 Cytotoxicity assessment

For the cytotoxicity assessment of mHER2_aHPF or hHER2_aHPF nanoparticles, RAW264.7 or DC2.4 cells were seeded into 6-well plates at a density of 5 × 10^5^ cells per well and incubated for 24 h. 200 μg/mL of mHER2_aHPF or hHER2_aHPF nanoparticles were added to each well and further incubated for 24 h or 48 h. Cells from different treatments were stained with the Annexin V-FITC/PI kit (Keygen Biotech, China) and detected by flow cytometry. In flow cytometry analysis, the cell populations were identified as follows: Annexin V^−^/PI^−^ represented for viable cells, Annexin V^+^/PI^−^ for early apoptosis, Annexin V^+^/PI^+^ for late apoptosis, and Annexin V^−^/PI^+^ for necrotic cells.

### 2.13 Cellular uptake assay

The gene encoding RFP protein was inserted into an expression plasmid 3.1-Intron-SP-WH-m/hHER2-RFP-ST003-IRES-GFP-WPRE, and the WH-m/hHER2-RFP-ST003 protein was expressed and purified using a mammalian cell expression system as described above. After incubating WH-m/hHER2-RFP-ST003 protein with aHPF nanoparticles, the conjugate samples were analyzed by SEC. The eluted fractions were collected and concentrated, and finally the mHER2/hHER2-RFP_aHPF nanoparticles were obtained. DC2.4 cells were seeded in 12-well plates at a density of 2 × 10^5^ cells per well and cultured overnight. 1 nmol of mHER2-RFP, hHER2-RFP, mHER2-RFP_aHPF, and hHER2-RFP_aHPF proteins were added to each well, respectively, with PBS serving as the negative control. After incubating for 6 h, cells were harvested, washed three times with PBS and then analyzed by Cytoflex S flow cytometer (Beckman Coulter, USA) to quantify the percentages of RFP-positive cells.

### 2.14 BMDC maturation assay

Mouse bone marrow cells were harvested from tibias and femurs of BALB/c mice and treated with red blood cell lysis buffer. Cells were then cultured in RPMI 1640 medium supplemented with 10% FBS, 20 ng/mL GM-CSF, and 10 ng/mL IL-4. Half the medium was replaced every 2 days, and the induced bone marrow-derived dendritic cells (BMDCs) were harvested on Day 7. BMDCs were incubated with 1 nmol of aHPF, mHER2, hHER2, mHER2_aHPF, and hHER2_aHPF proteins, respectively. After incubation for 24 h, cells and supernatants were collected, respectively. Cells were stained with anti-CD11c-PerCP-Cy5.5, anti-CD80-APC, and anti-CD86-APC-Cy7 antibodies for 30 min. The proportions of mature BMDCs were analyzed by flow cytometry to quantify the percentages of CD11c^+^CD80^+^CD86^+^ cells. The secretion levels of inflammatory cytokines including IL-6 (EM0004, Huabio, China) and TNF-α (EMC102a.96, NeoBioscience, China) within supernatants were quantified using commercial enzyme-linked immunosorbent assay (ELISA) kits according to the manufacturers' instructions.

### 2.15 Hemolysis assay

Whole blood collected from mice was centrifuged at 2800 rpm for 10 min to isolate red blood cells. The red blood cell pellet was washed three times with PBS and incubated with 200, 500, 1,000, 2,500, 5,000, or 10,000 μg/mL of mHER2_aHPF or hHER2_aHPF nanoparticles at 37°C for 1 h. For control groups, PBS-treated and ddH_2_O-treated red blood cells served as the negative and positive controls, respectively. After the incubation, the mixtures were centrifuged at 10,000 g for 10 min, and the absorbance value of the supernatant was measured at 540 nm. The hemolysis ratio was calculated as the percentage relative to the ddH_2_O positive control, which was designated as 100%.

### 2.16 Quantitative real-time PCR (RT-qPCR) analysis

DC2.4 cells were seeded in 12-well plates at a density of 2 × 10^5^ cells per well and allowed to adhere overnight. Cells were then treated with PBS, CpG (30 μg), FNP (60 μg), or FNP (60 μg) combined with CpG (30 μg) for 24 h. Total RNA was extracted using the EZ-press RNA Purification Kit (B0004D, EZBioscience), and 1 μg of total RNA was reverse-transcribed into cDNA using the StarScript Pro All-in-One RT Mix Reagent Kit (A240-10, GeneStar). Real-time quantitative PCR was performed on a QuantStudio Real-Time PCR system using RealStar Fast SYBR qPCR Mix (A304, GeneStar) (Table S1). The relative mRNA expression levels of target genes were calculated using the comparative 2^−ΔΔCt^ method with mouse *Gapdh* as the internal control.

### 2.17 Detection of HER2 antigen-specific IgG antibody

An ELISA was performed to detect HER2-specific IgG antibody in the sera of immunized mice. 3 μg/mL of mouse or human HER2 protein was coated on 96-well ELISA plates and incubated overnight at 4°C. After washing with PBS/T for 3 times, the plates were blocked with PBS containing 5% non-fat milk for 1 h. After washing 3 times with PBS/T buffer, serum samples were 10-fold serially diluted in PBS and added to each well, followed by incubation at 37°C for 1 h. 100 μL of horseradish peroxidase (HRP)-conjugated goat anti-mouse secondary antibodies (diluted 1:10,000 in PBS buffer) were added to each well, with incubation at 37°C for 1 h. After washing with PBST for 5 times, TMB chromogenic solution was added, followed by dark incubation at room temperature for 15 min. Reactions were stopped by adding 0.1 mL of 1 M H_2_SO_4_, and absorbance values were read at 450 nm. The absorbance values were analyzed with non-linear regression to calculate the endpoint titer of each serum.

### 2.18 Antibody-dependent cellular phagocytosis assay

Heat-inactivated sera obtained from vaccinated mice were subjected to the antibody-dependent cellular phagocytosis (ADCP) assay. Briefly, 4T1 cells expressing mouse or human HER2 were incubated with 5-fold diluted sera for 12 h at 37°C. After being washed with PBS, cells were stained using the CellTrace Violet (CTV) Cell Proliferation Kit (Thermo Fisher, USA), followed by co-culturing with RAW264.7 mouse macrophage cell lines at an effector:target ratio of 1:1 (macrophage:tumor cell) for 2 h at 37°C. For Fc receptor blocking, RAW264.7 cells were pre-incubated with 2.5 μg/mL anti-mouse CD16/CD32 antibody (clone 93, 101319, BioLegend), which blocks FcγRIIB and FcγRIII, for 10 min at 4°C prior to co-culture. After the co-culture, all cells were harvested, washed three times with PBS, and then stained with PE-conjugated anti-mouse F4/80 antibodies (123110, BioLegend). The percentages of CTV^+^F4/80^+^ events were analyzed via FCM and presented as the proportions of tumor cell-phagocytosed macrophages.

### 2.19 Biodistribution of HER2_aHPF

mHER2, mHER2_aHPF, hHER2, and hHER2_aHPF were labeled with Sulfo-Cy7 NHS Ester (FD-CY7S01, Biolight Biotech) for 2 h, and free dye was removed by ultrafiltration. Equivalent Cy7 fluorescence across formulations was verified by UV-Vis spectroscopy prior to injection. Female BALB/c mice received 0.4 nmol of each labeled protein subcutaneously in the cervicodorsal region. At 0.5, 6, 12, and 24 h after injection, the heart, liver, spleen, lung, kidney, and draining lymph node were excised. *Ex vivo* fluorescence imaging was performed on a small-animal imaging system (BLT AniView100 Pro, Biolight Biotech) at excitation and emission wavelengths of 745 nm and 820 nm, respectively. The average radiance (p/s/cm^2^/sr) within each organ was determined by region-of-interest analysis.

### 2.20 Evaluation of HER2_aHPF nanoparticles immunogenicity

To evaluate whether freeze-thaw treatment affected vaccine immunogenicity, female BALB/c mice (*n* = 4 per group) were immunized with 1 nmol of mHER2_aHPF or hHER2_aHPF that had been subjected to 0, 1, 3, 5, 7, and 9 freeze-thaw cycles, administered on Days 0, 7, and 14. On Day 21, serum was collected and HER2-specific IgG endpoint titers were measured by ELISA.

To evaluate the immunological efficacy of vaccines *in vivo*, female BALB/c mice aged 6-8 weeks were subcutaneously immunized on Days 0, 7, and 14 with aHPF, mHER2/hHER2, and mHER2/hHER2_aHPF, respectively. All groups were co-administered an equal volume (50 μL) of adjuvant, consisting of 30 μg CpG and 60 μg FNP per dose. The nanoparticle vaccines (1 nmol per dose) were prepared in 50 μL of buffer (20 mM Tris-HCl, 50 mM NaCl, pH 7.5). All subcutaneous injections were administered in the scruff of the neck. The molar amounts of aHPF, mHER2/hHER2, and mHER2/hHER2_aHPF were kept consistent. On Day 21 post-immunization, blood samples were collected via orbital bleeding and serum was isolated for subsequent analysis. Antigen-specific IgG antibody titers in the serum were quantified using ELISA to evaluate the humoral immune response. Meanwhile, spleens and inguinal lymph nodes were harvested and processed into single-cell suspensions. The cellular immune response was assessed by flow cytometric analysis of splenic CD4^+^ and CD8^+^ T cell subpopulations, cytokine secretion profiles (IFN-γ and TNF-α), and maturation status of dendritic cells in the lymph nodes.

### 2.21 In vivo safety assessment

Female BALB/c mice were immunized with the indicated formulations (Mock, Adjuvant, mHER2_aHPF, and hHER2_aHPF) on Days 0, 7, and 14. On Day 20, whole blood and serum were collected. Major organs, including heart, liver, spleen, lungs, and kidneys, were harvested. The tissues were fixed in 4% paraformaldehyde (PFA) for 24 h, then dehydrated, embedded in paraffin, and sectioned at a thickness of 3-5 μm. Sections were deparaffinized, rehydrated, and stained with hematoxylin and eosin (H&E) according to standard protocols. Whole blood collected via the retro-orbital sinus was analyzed using an automated hematology analyzer for red blood cell (RBC) count (reference range 6.5-11.5 × 10^12^ cells/L), platelet (PLT) count (400-1600 × 10^9^ cells/L), hemoglobin (HGB) concentration (110-165 g/L), and hematocrit (HCT) percentage (35-55%). Serum was obtained by centrifugation at 2800 rpm for 10 min. Hepatic function was assessed by measuring alanine aminotransferase (ALT; 10.06-96.47 U/L), aspartate aminotransferase (AST; 36.31-235.48 U/L), alkaline phosphatase (ALP; 22.52-474.35 U/L), total bilirubin (TBIL; 6.09-53.06 μmol/L), gamma-glutamyl transferase (γ-GT; 0-7.78 U/L), and total bile acids (TBA; 0-8.51 μmol/L). Renal function was assessed by measuring urea (UREA; 3.9-12.4 mmol/L) and creatinine (CREA; 10.91-85.09 μmol/L). All biochemical assays were performed using an automatic chemistry analyzer with enzymatic colorimetric methods. Serum levels of IL-6 and TNF-α were quantified by ELISA according to the manufacturer's instructions.

### 2.22 Tumor model and immunization

For evaluation of the preventive efficacy of mHER2 nanoparticle vaccines, female BALB/c mice were randomly divided into four experimental groups. Mice were immunized three times with adjuvant, 1 nmol aHPF, 1 nmol mHER2, and 1 nmol mHER2_aHPF, respectively. Vaccine formulations (1 nmol in 50 μL of Tris-HCl buffer) were injected subcutaneously in the scruff of the neck, with the adjuvant solution (50 μL) co-administered at the same site. All these vaccine formulations contained the same adjuvant formulation, which consisted of 30 μg CpG and 60 μg FNP. On Day 15, mice were challenged by subcutaneous injection of 1 × 10^5^ of 4T1-mHER2-Luc cells in PBS into the right back. Tumor dimensions were measured every two days, and tumor volume was calculated using formula V = length × width^2^/2. Mice were euthanized when tumor volume reached 1000 mm^3^.

For the lung metastasis prevention model, BALB/c mice were randomized into six groups and treated with adjuvant, anti-PD-1/anti-CD47 antibodies, aHPF, mHER2, mHER2_aHPF, or combined treatment of mHER2_aHPF and anti-PD-1/anti-CD47 antibodies on Day −14, −7, and 0. Both anti-PD-1 and anti-CD47 antibodies (100 μg per dose) were administered intraperitoneally in 100 μL of PBS. About 8 × 10^5^ of 4T1-mHER2-Luc cells were administered to the mice via tail vein injection on Day 1. For the combined treatment of mHER2_aHPF and anti-PD-1/anti-CD47 group, Anti-PD-1 and anti-CD47 antibodies (100 μg per mouse for each injection) were injected intraperitoneally three times at two-day intervals. Bioluminescence imaging utilizing the *IVIS* imaging system was conducted on Days 3, 5, 8, and 15 to monitor lung metastasis. For *in vivo* bioluminescence imaging, mice were anesthetized with isoflurane and subsequently administered D-luciferin potassium salt (150 mg/kg) via intraperitoneal injection. At 10 min post-injection, bioluminescent signals were acquired using the AniView100pro *in vivo* imaging system (Guangzhou Biolight Biotechnology Co., Ltd., China) under sustained isoflurane anesthesia.

For the combined treatment model involving both the mHER2 nanoparticle vaccine and the immune checkpoint blockade (ICB) therapy, female BALB/c mice were inoculated subcutaneously with 1 × 10^5^ of 4T1-mHER2-Luc cells in the right back. After tumor establishment, mice were randomized into six groups and treated as follows: mice received subcutaneous injections of adjuvant control, 1 nmol aHPF, 1 nmol mHER2, 1 nmol mHER2_aHPF, or 1 nmol mHER2_aHPF combined with ICB antibodies every 7 days for a total of three doses. Anti-PD-1 antibody (100 μg per mouse) was injected intraperitoneally on Days 6, 9, and 16, and anti-CD47 antibody (100 μg per mouse) was injected intraperitoneally on Days 12, 15, and 17. The combined treatment regimen involving the hHER2 nanoparticle vaccine and ICB therapy was implemented similarly, with the exception that antigens used in this model were derived from hHER2, and cancer cell lines employed were 4T1-hHER2-Luc cells.

A head-to-head comparison between hHER2_aHPF vaccination and trastuzumab was performed in the therapeutic setting. Female BALB/c mice were subcutaneously inoculated with 4T1-hHER2-Luc cells on Day 0 and assigned to three groups of four mice each. The Adjuvant group received CpG-FNP adjuvant only. The hHER2_aHPF group received 1 nmol hHER2_aHPF co-administered with CpG-FNP adjuvant via subcutaneous injection on Days 1, 7, and 14. The Trastuzumab group received intraperitoneal injections of trastuzumab (T9912, TargetMol) at 250 μg per mouse, equivalent to approximately 10 mg/kg for a 25-g mouse, on Days 2, 8, and 15. Tumor volume was monitored on Days 0, 3, 5, 7, 9, 11, 14, 16, 18, and 20, and all mice were euthanized on Day 20.

### 2.23 HER2 expression detection in tumor tissues

For analysis of HER2 expression in tumor tissues, female BALB/c mice were immunized with mHER2_aHPF, hHER2_aHPF, or adjuvant on Days 0, 7, and 14, followed by subcutaneous challenge with 4T1-mHER2 or 4T1-hHER2 cells on Day 15. Mice were euthanized on Day 37, and tumor tissues were harvested. Tissues were minced and digested with collagenase I (1.0 mg/mL) (BS163-100 mg, Biosharp, China), DNase I (0.1 mg/mL) (D8071, Solarbio, China), and hyaluronidase (0.1 mg/mL) (abs47014926, Absin, China) at 37°C for 30 min. After red blood cell lysis with ACK buffer (555899, BD Biosciences, USA), the cell suspension was filtered through a 70-μm strainer to obtain single cells. Cells were first stained with anti-CD45-APC-Cy7 (clone 30-F11, 103116, BioLegend) and Fixable Viability Dye eFluor 506 (65-0866-14, Thermo Fisher Scientific). Following fixation and permeabilization, intracellular HER2 was detected with an anti-HER2 primary antibody (ab214275, Abcam, 1:60) for 30 min, followed by an AF647-conjugated secondary antibody (1:1000) for 30 min. The stained cells were analyzed by flow cytometry. HER2 expression was assessed on gated CD45^−^ viable cells. The 4T1-mHER2 or 4T1-hHER2 cell line served as positive control, and unstained cells as negative control.

### 2.24 Intracellular cytokine staining

Splenic and tumor tissues were harvested from immunized mice. Tumor tissues were cut into small fragments and incubated with a digestive enzyme cocktail containing 1.0 mg/mL of collagenase I, 0.1 mg/mL of DNase I, and 0.1 mg/mL of hyaluronidase at 37°C for 0.5 h. Following digestion, red blood cells were removed by incubation in ACK lysis buffer, and the resulting cell suspension was filtered through a 70 μm strainer to obtain single-cell preparations. For splenic tissues, single-cell suspensions were generated via mechanical dissociation through a 70 μm cell strainer. Cells were adjusted to a density of 1 × 10^6^ cells/well in 12-well plates and stimulated with mouse or human HER2 peptide pools, supplemented with 1 μg/mL of anti-CD28 antibodies (102116, BioLegend, USA) for 6 h. About 1 μg/mL of Brefeldin A (S7046, Selleck Chemicals, USA) was added during stimulation to block cytokine secretion. After stimulation, tumor-derived single-cell suspensions were stained with fluorescent dye-conjugated anti-mouse antibodies including anti-CD3-FITC, anti-CD4-AF700, anti-CD8α-PE-Cy7, and anti-CD45-APC-Cy7. Splenocytes were stained with anti-CD3-FITC, anti-CD4-AF700, and anti-CD8α-PE-Cy7 antibodies. Cells were subsequently fixed and permeabilized using a commercial intracellular staining kit (554714, BD Biosciences, USA) according to the manufacturer's instructions, followed by additional staining with anti-IFNγ-BV421, anti-TNF-α-APC, and anti-Granzyme B-PE antibodies. Stained cells were acquired on a flow cytometer and analyzed using FlowJo software.

### 2.25 In vivo immune memory analysis

Splenic tissues were harvested and single-cell suspensions prepared by mechanical dissociation, red blood cell lysis, and filtration through a 70 μm cell strainer as described above. Cells were stained with mouse antibodies including anti-CD3-FITC, anti-CD4-AF700, anti-CD8α-PE, anti-CD62L-PE-Cy7, and anti-CD44-BV650 antibodies. Stained samples were analyzed by flow cytometry. The CD3^+^/CD8^+^/CD44^+^/CD62L^−^ splenocytes were gated and represented as CD8^+^ effector-memory T (T_EM_) cells.

### 2.26 Immunosuppressive cell and degranulating CD8^+^ T cells analysis

The prepared single-cell suspensions from tumor and splenic tissues were stained with anti-CD3-FITC, anti-CD4-AF700, anti-CD45-APC-Cy7, anti-Foxp3-PE, and anti-CD25-APC antibodies. The CD45^+^/CD3^+^/CD4^+^/CD25^+^/Foxp3^+^ cells were gated and represented Tregs. Another proportion of cells was stained with anti-CD3-AF700, anti-CD8α-PE, anti-Gr-1-FITC, anti-CD45-APC-Cy7, anti-CD11b-PE-Cy7 and anti-CD107a-BV421 antibodies. The CD45^+^/CD11b^+^/Gr-1^+^ cells were gated and represented MDSC cells, while the CD45^+^/CD3^+^/CD8^+^/CD107a^+^ cells were analyzed and represented Degranulating CD8^+^ T cells.

### 2.27 Statistical analysis

Statistical information, including sample sizes, mean values, standard errors of the mean (SEM), *P* values, and statistical tests, has been shown in figures or figure legends. All experimental data were presented as the mean ± SEM, with each experiment independently repeated at least 3 times. Student's *t*-tests were conducted for comparisons between two groups, while ANOVA analyses were performed for comparisons between more than two groups. A *P* value of < 0.05 was statistically significant and represented as a single asterisk (*). A *P* value of < 0.01 was considered highly significant and represented as double asterisks (**). A *P* value of < 0.001 was considered as extremely significant and represented as triple asterisks (***).

## 3 Results

### 3.1 Construction and characterization of multitargeting HER2 nanoparticle vaccines

**Fig. 1:**
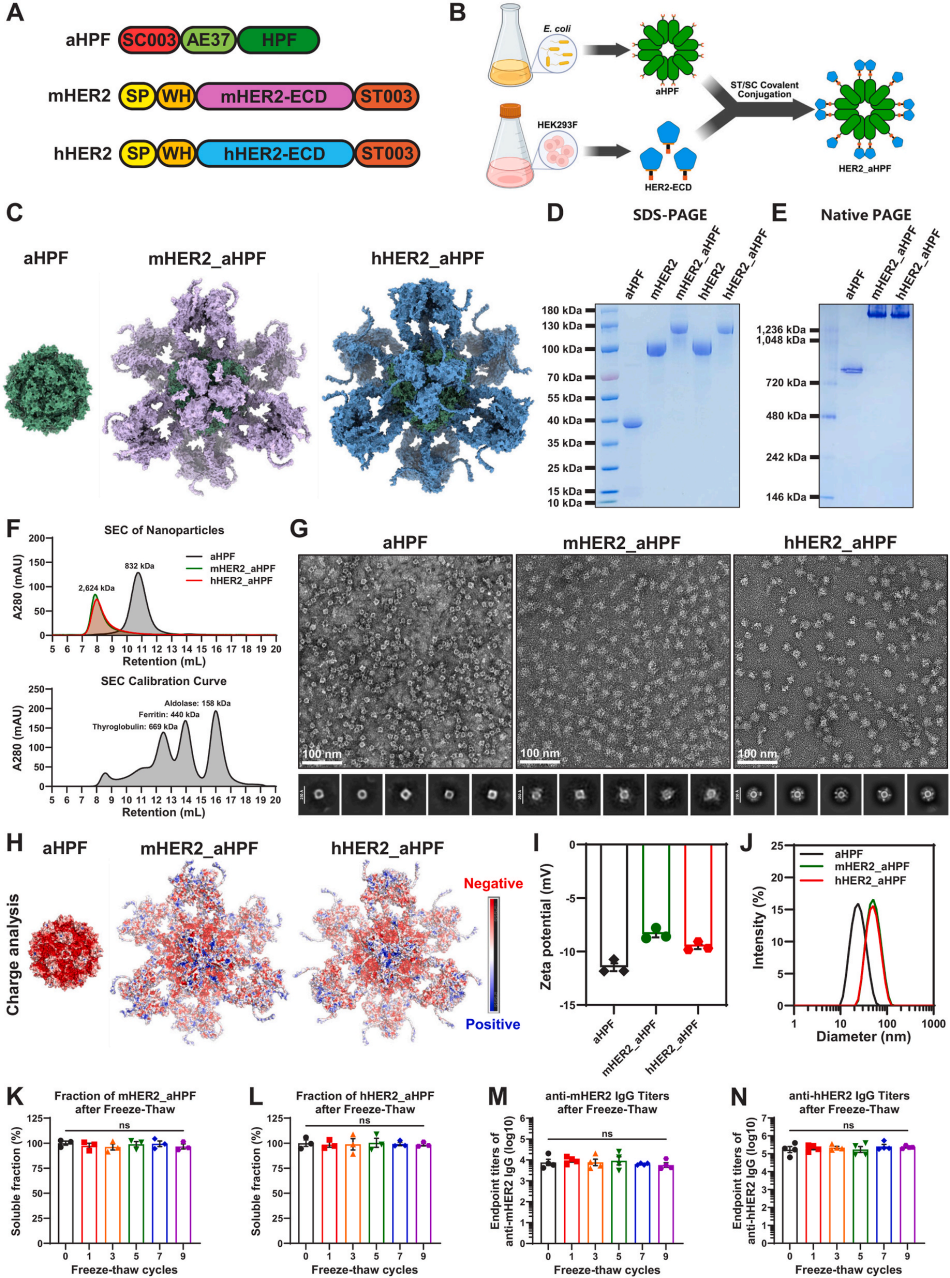
**The construction of ferritin nanoparticle vaccines against HER2-positive breast cancer.** (A) Schematic illustrations of vaccine components. aHPF: *Helicobacter pylori* ferritin (HPF) conjugated with SpyCatcher003 (SC003) and AE37 peptide. mHER2: the extracellular domain (ECD) of mouse HER2 tagged with signal peptide (SP), WH peptide, and SpyTag003 (ST003). hHER2: the ECD of human HER2 tagged with SP, WH peptide and ST003. (B) Schematic of nanoparticle vaccine production. aHPF nanoparticle cores were expressed in *E. coli*, while HER2 antigens were expressed by HEK293F cells and purified from supernatants. Purified aHPF and HER2 proteins were covalently conjugated via the ST/SC system to generate assembled HER2_aHPF nanoparticle vaccines. (C) 3D surface renderings of aHPF, mHER2_aHPF, and hHER2_aHPF. aHPF was colored green. mHER2 was colored magenta-pink, while hHER2 was colored cyan-blue. (D) SDS-PAGE analysis of aHPF, mHER2, mHER2_aHPF, hHER2, and hHER2_aHPF. (E) Native PAGE analysis of aHPF, mHER2_aHPF, and hHER2_aHPF. (F) Size-exclusion chromatography (SEC) profiles of purified aHPF, mHER2_aHPF, and hHER2_aHPF on a Superose 6 Increase 10/300 GL column. Absorbance at 280 nm (A280) was monitored. A calibration curve was generated using protein molecular weight standards (aldolase, 158 kDa; ferritin, 440 kDa; thyroglobulin, 669 kDa). The estimated molecular weights of aHPF, mHER2_aHPF, and hHER2_aHPF were approximately 832, 2,624, and 2624 kDa, respectively. (G) Representative negative staining images (scale bar, 100 nm) and two-dimensional (2D) class averages (scale bar, 250 Å) of aHPF, mHER2_aHPF, and hHER2_aHPF. A total of 1,884, 3,705, and 7387 particles were automatically picked for aHPF, mHER2_aHPF, and hHER2_aHPF, respectively. 2D class averages were generated using cryoSPARC. (H) Electrostatic surface renderings of aHPF, mHER2_aHPF, and hHER2_aHPF. Negative patches (red) and positive patches (blue) were highlighted and repeated according to icosahedral symmetry. (I) Zeta potential analysis of aHPF, mHER2_aHPF, and hHER2_aHPF in PBS. (J) Hydrodynamic diameters of aHPF, mHER2_aHPF, and hHER2_aHPF, measured by dynamic light scattering (DLS) in PBS. (K-L) Nanoparticle vaccines were subjected to 1 to 9 cycles of freeze-thaw treatment, with temperature transitioning from −80°C to 37°C. Percentages of soluble fractions of mHER2_aHPF (K) or hHER2_aHPF (L) were determined by quantifying the amounts of soluble proteins and then normalizing them to the input. (M − N) Retention of nanoparticle vaccine immunogenicity after freeze-thaw cycles. Female BALB/c mice (*n* = 4 per group) were immunized with mHER2_aHPF or hHER2_aHPF that had undergone 0, 1, 3, 5, 7, and 9 freeze-thaw cycles, respectively. Serum samples were collected to measure the endpoint titers of specific anti-mHER2 (M) or anti-hHER2 IgG (N) via enzyme-linked immunosorbent assay (ELISA). Data in (I) and (K-N) were shown as mean ± SEM. *P* values were calculated by one-way ANOVA with Tukey's multiple comparisons tests. ns, not significant.

Tumor vaccines that establish an immune activation system through dual-axis coordination of both cellular and humoral immune responses could more effectively combat tumors and prevent immune escape. Therefore, we designed a multitargeting nanoparticle vaccine by incorporating both T-cell and B-cell epitopes of HER2 antigens. A CD4^+^ T-cell epitope AE37, which is derived from the intracellular domain (ICD) of HER2, was expressed in-frame with *Helicobacter pylori* ferritin (HPF) to construct the aHPF (AE37 peptide-HPF fusion nanoparticle core) (Fig. 1A) [22]. We also attempted to incorporate a CD8^+^ T-cell epitope GP2 derived from the transmembrane domain (TMD) of HER2 on HPF [21]. However, due to the highly hydrophobic nature of GP2, the resulting GP2-conjugated HPF was unable to form 24-meric nanoparticles. Moreover, the GP2 sequence diverges between mouse and human HER2, which would complicate cross-species evaluation of vaccine efficacy. Given these considerations, GP2 was not pursued further in this study. The full extracellular domain (ECD) of mouse or human HER2, which contained both B-cell epitopes targeted by monoclonal antibodies (mAbs) such as trastuzumab and pertuzumab and the CD8^+^ T-cell epitope E75, was expressed as the outer antigen of the nanoparticle vaccine (Fig. 1A) [20,24]. Sequence alignment and structural analysis confirmed that both the E75 and AE37 epitopes are completely identical between mouse and human HER2 proteins, supporting the translational relevance of evaluating these vaccines in murine models expressing the human antigen (Fig. S1A and S1B). The secretory signal peptide (SP) was expressed in-frame with HER2-ECD. To enhance the antigen uptake of vaccines by dendritic cells (DCs), a DC Clec9a-targeting WH peptide was conjugated at the N-terminal of HER2-ECD as well [50]. Meanwhile, SpyCatcher003 (SC003) and SpyTag003 (ST003) were respectively expressed in-frame with aHPF and mHER2/hHER2 to covalently conjugate WH-HER2-ECD to the surface of aHPF [49]. aHPF nanoparticle cores and HER2 antigens were expressed in *Escherichia coli* (*E. coli*) and HEK293F cells, respectively, followed by *in vitro* affinity purification and ST/SC conjugation to finalize the HER2_aHPF nanoparticle vaccines (Fig. 1B). The *in vitro* conjugation assays demonstrated that ST003-tagged aHPF nanoparticles were fully conjugated with SC003-tagged HER2 antigens at a molar ratio of aHPF to HER2 of 1:3 (Fig. S2A–S2C). Time-course analysis at the 1:3 ratio showed that the conjugation reaction proceeded rapidly, with mHER2_aHPF and hHER2_aHPF reaching near-complete conjugation within 120 min (Fig. S2D–S2G).

Theoretically, 24 copies of mouse or human HER2 antigens could be simultaneously conjugated to the outer surface of 24-meric aHPF (Fig. 1C). To verify the conjugation efficiency, both SDS-PAGE and native PAGE were utilized. The SDS-PAGE results indicated that HER2_aHPF migrated as a single band to the 130 kDa position, which was consistent with the sum of molecular weights of aHPF and HER2 (Fig. 1D). The native PAGE results revealed that the native conformational HER2_aHPF migrated beyond 1236 kDa, while aHPF migrated between 720 kDa and 1048 kDa (Fig. 1E). Size-exclusion chromatography (SEC) on a Superose 6 Increase column calibrated with protein standards showed that aHPF eluted at 10.73 mL, corresponding to an estimated molecular weight of approximately 832 kDa (Fig. 1F). The mHER2_aHPF and hHER2_aHPF conjugates eluted at 7.87 mL and 7.98 mL, respectively, with estimated molecular weights of approximately 2624 kDa each. The substantial shifts in retention volume relative to unconjugated aHPF confirmed a large increase in molecular size upon HER2 conjugation. Further negative staining characterization with automated particle picking and two-dimensional (2D) classification using cryoSPARC confirmed the structural homogeneity and monodispersity of nanoparticles, with average diameters of 15 nm and 23 nm for aHPF and HER2_aHPF, respectively (Fig. 1G). aHPF nanoparticle cores displayed hollow spherical structures, while HER2-conjugated nanoparticles exhibited crown-like morphologies with electron-dense peripheral projections, which indicated the successful surface arrangement of HER2 antigens on these nanoparticles. Both mouse and human HER2 nanoparticle vaccines were predominantly negatively charged through electrostatic surface renderings (Fig. 1H). Zeta potential measurements in PBS yielded values of −11.47 ± 0.62 mV, −8.38 ± 0.52 mV, and −9.54 ± 0.40 mV for aHPF, mHER2_aHPF, and hHER2_aHPF, respectively (Fig. 1I). The average hydrodynamic diameters of aHPF, mHER2_aHPF, and hHER2_aHPF measured by dynamic light scattering (DLS) in PBS were 23.88, 50.79, and 50.79 nm, respectively (Fig. 1J). Both conjugates exhibited essentially identical hydrodynamic diameters, consistent with their similar molecular weights. These values were larger than those measured by negative staining because the hydrodynamic diameter represents the effective size of particles along with associated solvent layers. These negatively charged nanoparticles could facilitate lymphatic drainage and lymph node targeting of vaccines by preventing non-specific cellular uptake in peripheral tissues, consequently safeguarding the WH peptide-guided specific internalization and presentation by DCs [53].

As an additional quality attribute relevant to clinical translation, endotoxin levels in aHPF, mHER2_aHPF, and hHER2_aHPF were measured using a chromogenic Tachypleus amebocyte lysate (TAL) assay. All three preparations contained endotoxin levels below the pharmacopoeial limit for parenteral products (Fig. S2L). To evaluate the stability of HER2_aHPF nanoparticle vaccines and verify their feasibility for clinical application, mHER2_aHPF and hHER2_aHPF were tested under different storage conditions, including repeated freeze-thaw cycles and various storage durations at different temperatures. Both mHER2_aHPF and hHER2_aHPF underwent 1 to 9 cycles of freeze-thaw treatment, during which the temperature transitioned from −80°C to 37°C. The results demonstrated that these nanoparticle vaccines maintained stable solubility without any aggregation after 1 to 9 cycles of freeze-thaw, which was comparable to freshly prepared vaccines without freeze-thaw (Fig. 1K and L). To determine whether the vaccines retained immunogenicity after freeze-thaw stress, female BALB/c mice were immunized with 1 nmol of mHER2_aHPF or hHER2_aHPF that had been subjected to 0, 1, 3, 5, 7, and 9 freeze-thaw cycles, administered on Days 0, 7, and 14. On Day 21, serum was collected and HER2-specific IgG endpoint titers were measured by enzyme-linked immunosorbent assay (ELISA). Anti-mHER2 and anti-hHER2 IgG titers were comparable across all freeze-thaw conditions, with no significant differences between the freshly prepared vaccines and those subjected to up to 9 cycles (Fig. 1M and N, S2M, and S2N). Furthermore, the thermal stability of these nanoparticle vaccines was evaluated by storing HER2_aHPF at −80°C for 2 months, −20°C for 1 month, and 4°C for 1 week, respectively. The SDS-PAGE analysis followed by densitometric quantification showed that intact protein accounted for over 97% of the total band intensity across all storage conditions, confirming that both mHER2_aHPF and hHER2_aHPF maintained structural integrity without detectable degradation (Fig. S2H–S2K). Collectively, both mHER2_aHPF and hHER2_aHPF nanoparticle vaccines demonstrated high thermal stability and freeze-thaw resistance, fulfilling the fundamental requirements for the long-term preservation of clinical preparations and cold-chain transportation.

### 3.2 HER2 nanoparticle vaccines enhance antigen uptake and BMDC maturation

**Fig. 2:**
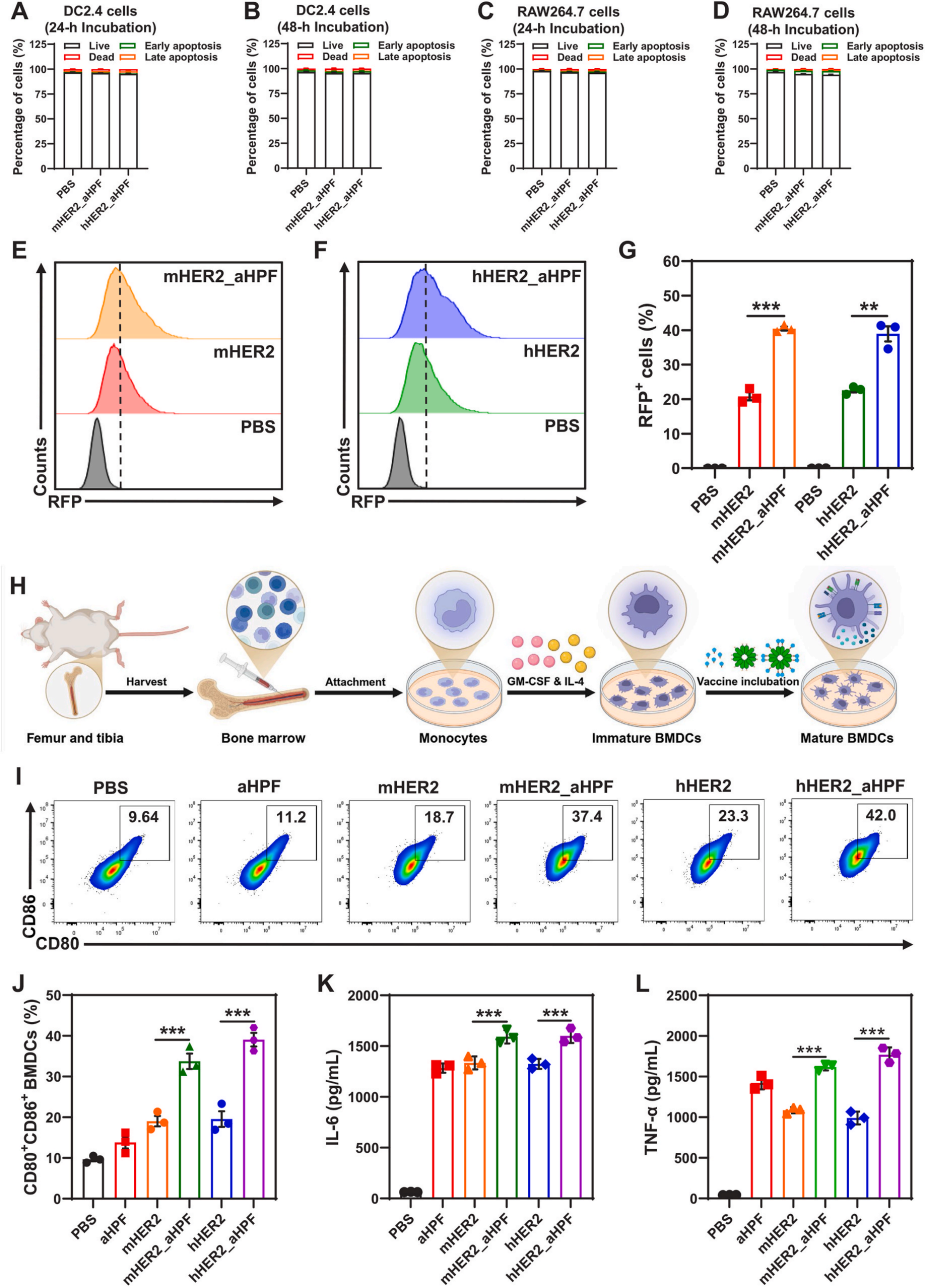
**HER2 nanoparticle vaccines promote both antigen uptake and maturation of BMDCs.** (A-D) DC2.4 cells (A-B) and RAW264.7 cells (C-D) were incubated with 200 μg/mL of mHER2_aHPF or hHER2_aHPF for 24 h (h) (A, C) or 48 h (B, D), followed by staining with Annexin V-FITC and PI. The percentages of live (Annexin V^−^/PI^−^), dead (Annexin V^−^/PI^+^), early apoptotic (Annexin V^+^/PI^−^), and late apoptotic (Annexin V^+^/PI^+^) cells were quantified by flow cytometry (FCM) for each group. Treatment with PBS served as a negative control. (E-F) DC2.4 cells were incubated with 1 nmol of RFP-tagged mHER2, mHER2_aHPF, hHER2, or hHER2_aHPF for 6 h, followed by detection RFP-positive cells by FCM. (G) The percentages of RFP-positive cells were quantified. PBS treatment was set as the negative control. (H) Schematic of bone marrow-derived dendritic cell (BMDC) maturation assay. Briefly, mouse bone marrow cells derived from femur and tibia of BALB/c mice were cultured in conventional RPMI 1640 medium supplemented with 20 ng/mL GM-CSF and 10 ng/mL IL-4 for 7 days, followed by incubation with 1 nmol aHPF, mHER2, hHER2, mHER2_aHPF, or hHER2_aHPF for another 24 h. Mature BMDCs were quantified via FCM, while inflammatory cytokines within supernatants were quantified by ELISA. (I) Representative FCM plots depicting BMDCs after treatment with different antigens. (J) Statistical quantification of CD80^+^CD86^+^ mature BMDCs. (K-L) The concentrations of inflammatory cytokines including IL-6 (K) and TNF-α (L) within supernatants were quantified by ELISA. Data in (A-D), (G), and (J-L) were presented as mean ± SEM. *P* values were calculated by one-way ANOVA with Tukey's multiple comparisons tests. **P < 0.01, ***P < 0.001.

The initiation of adaptive immune responses relies on safe and efficient antigen-presentation by antigen-presenting cells (APCs), such as dendritic cells (DCs) and macrophages, which internalize, process, and present antigens to T cells [54]. Therefore, prior to systemic immunological evaluation, we first investigated the potential cytotoxicity of mHER2_aHPF and hHER2_aHPF on APCs. DC2.4 mouse dendritic cells were incubated with 200 μg/mL of mHER2_aHPF or hHER2_aHPF for 24 h (h) or 48 h, followed by staining with Annexin V and PI to probe apoptotic cells. The flow cytometry (FCM) analysis revealed that there were no significant differences in the proportions of live (Annexin V^−^/PI^−^), dead (Annexin V^−^/PI^+^), early apoptotic (Annexin V^+^/PI^−^), and late apoptotic (Annexin V^+^/PI^+^) cells among the PBS, mHER2_aHPF, and hHER2_aHPF treatments at either time point (Fig. 2A and B, S3A, and S3B). Similarly, the treatment of RAW264.7 mouse macrophage cells with HER2 nanoparticle vaccines did not induce significant apoptosis or necrosis (Fig. 2C and D, S3C, and S3D). Dose-dependent hemolysis was evaluated by incubating red blood cells with mHER2_aHPF or hHER2_aHPF at concentrations ranging from 200 to 10,000 μg/mL. No detectable hemolysis was observed at any concentration tested (Fig. S3E and S3F). These results demonstrated that HER2 nanoparticle vaccines exhibited high biocompatibility.

The WH peptide presented on antigens and the nanoscale scaffold of HPF have been shown to facilitate antigen uptake by DCs, leading to efficient DC maturation [33,50]. To evaluate whether HER2 nanoparticle vaccines could be more effectively internalized by DCs, DC2.4 cells were incubated with 1 nmol of RFP-tagged mHER2, mHER2_aHPF, hHER2, and hHER2_aHPF for 6 h, respectively, followed by FCM to quantify percentages of RFP-positive cells (Fig. 2E and F). The FCM data revealed that both mHER2 and hHER2 were effectively internalized by DC2.4 cells, while mHER2_aHPF and hHER2_aHPF exhibited higher uptake efficiencies compared to the monomeric antigens (Fig. 2G). These observations indicated that the WH peptide and HPF nanoparticles effectively promoted antigen internalization by APCs. We further investigated whether HER2 nanoparticle vaccines could facilitate the maturation of APCs. Mouse bone marrow-derived monocytes were isolated and enriched from tibias and femurs of BALB/c mice, followed by GM-CSF and IL-4 treatments to induce the generation of bone marrow-derived dendritic cells (BMDCs) (Fig. 2H). Subsequently, these immature BMDCs were incubated with 1 nmol of aHPF, mHER2, hHER2, mHER2_aHPF, and hHER2_aHPF, respectively. The percentages of mature BMDCs were quantified by FCM at 24 h after incubation (Fig. S3G). The FCM results showed that both mHER2_aHPF and hHER2_aHPF treatments led to higher proportions of CD80^+^CD86^+^ mature BMDCs compared to their respective monomeric forms (Fig. 2I and J). Besides the upregulation of CD80 and CD86 co-stimulatory molecules, mature DCs also secrete IL-6 and TNF-α cytokines to synergize with co-stimulatory molecules for optimal T-cell activation [55]. Upon incubating BMDC with mHER2_aHPF and hHER2_aHPF, the secretion of these cytokines was significantly increased compared to that with monomeric HER2 antigens (Fig. 2K and L). Above all, these results demonstrated that HER2 nanoparticle vaccines effectively promoted the antigen uptake and functional maturation of DCs without inducing significant cytotoxicity.

### 3.3 Fullerenol functions as a potent immunoadjuvant for nanoparticle vaccines

**Fig. 3:**
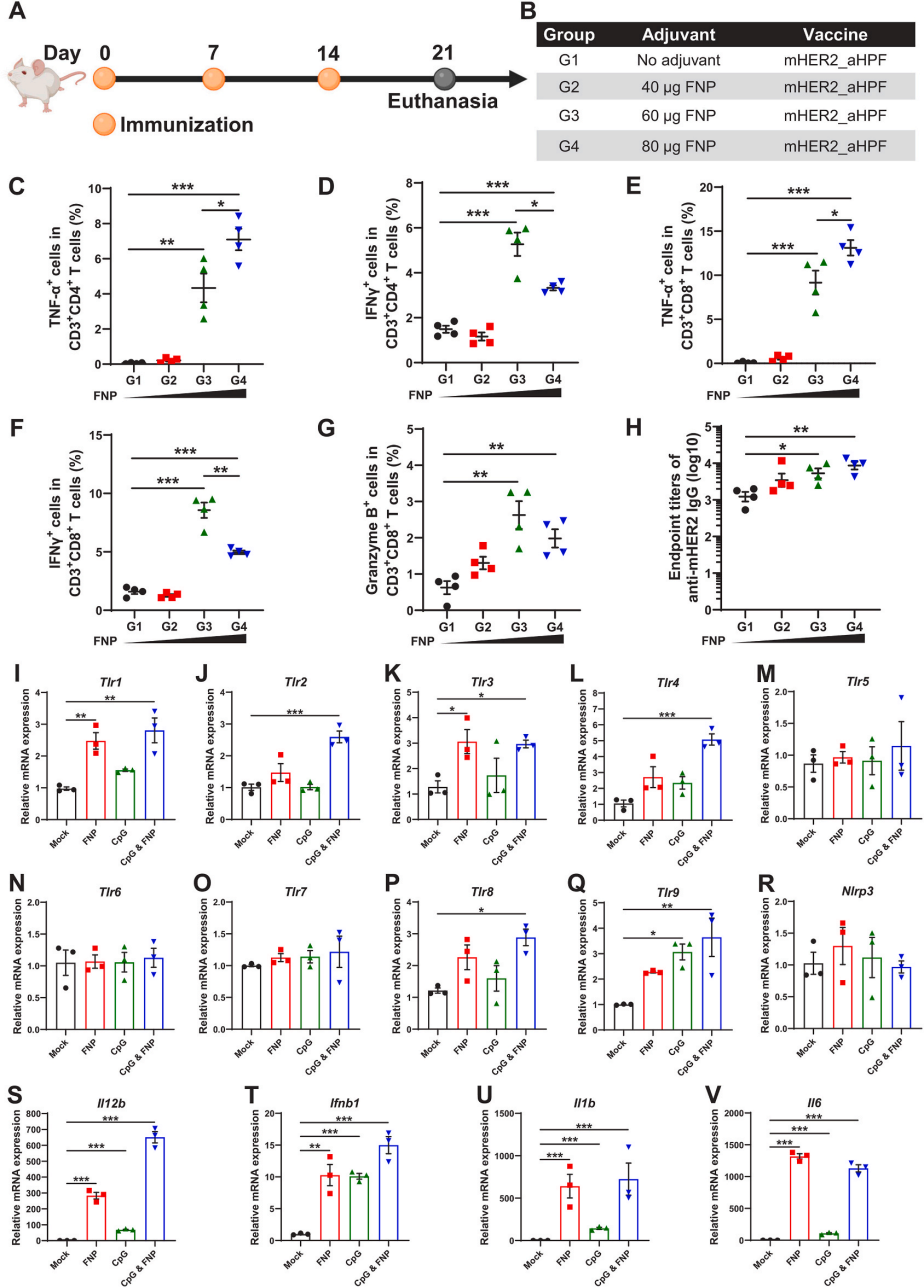
**Fullerenol enhances the immune responses elicited by nanoparticle vaccines.** (A) Schematic of vaccination timeline in female BALB/c mice (*n* = 4 per group). Each mouse was vaccinated on Day 0 (prime), 7 (boost), and 14 (booster), followed by euthanasia on Day 21. Splenocytes were processed for functional lymphocyte quantification, while sera were collected for antibody titer determination. (B) Experimental vaccination groups. Group 1 (G1): 10 μg of mHER2_aHPF without any adjuvant. G2: 10 μg of mHER2_aHPF adjuvanted with 40 μg of fullerenol nanoparticle (FNP). G3: 10 μg of mHER2_aHPF adjuvanted with 60 μg of FNP. G4: 10 μg of mHER2_aHPF adjuvanted with 80 μg of FNP. (C-D) Antigen-specific polyfunctional lymphocytes within each group were stimulated with mouse HER2 peptide pools, followed by intracellular cytokine staining (ICCS). Percentages of TNF-α^+^ (C) and IFNγ^+^ (D) CD4^+^ T cells were analyzed via FCM and normalized to total numbers of CD3^+^CD4^+^ T cells. (E-G) Percentages of antigen-specific TNF-α^+^ (E), IFNγ^+^ (F), and Granzyme B^+^ (G) CD8^+^ T cells were analyzed as in (C-D) and normalized to total numbers of CD3^+^CD8^+^ T cells. (H) Endpoint titers of mouse HER2-specific IgG within each serum were quantified via ELISA. (I-V) DC2.4 cells were treated with PBS, CpG (30 μg), FNP (60 μg), or the CpG-FNP combination for 24 h, followed by RT-qPCR quantification of the mRNA expression of *Tlr1* (I), *Tlr2* (J), *Tlr3* (K), *Tlr4* (L), *Tlr5* (M), *Tlr6* (N), *Tlr7* (O), *Tlr8* (P), *Tlr9* (Q), *Nlrp3* (R), *Il12b* (S), *Ifnb1* (T), *Il1b* (U), and *Il6* (V), normalized to mouse *Gapdh*. Data in (C-V) were presented as mean ± SEM. *P* values were calculated by one-way ANOVA with Tukey's multiple comparisons tests. *P < 0.05, **P < 0.01, ***P < 0.001.

Previously, fullerenol nanoparticles (FNPs) have been shown to induce the disruption of actin cytoskeleton assembly in cancer cells and the exposure of the “eat me” signal calreticulin on the surface of cancer cells, thereby inhibiting tumor growth and lung metastasis of breast cancer [52,56]. However, it remains unknown whether FNP can also function as an immunostimulatory adjuvant for vaccines. Therefore, we immunized female BALB/c mice with FNP-adjuvanted mHER2_aHPF nanoparticle vaccines. Each mouse was administered three doses of vaccines on Day 0 (prime), 7 (boost), and 14 (booster), followed by euthanasia on Day 21 for the quantification of immune responses (Fig. 3A). Mice administered with only 10 μg of mHER2_aHPF vaccine without any adjuvant were designated as the control group (G1), while mice in G2, G3, and G4 groups were administered with 40, 60, and 80 μg of the FNP-adjuvanted nanoparticle vaccines, respectively (Fig. 3B). Splenocytes isolated from euthanized mice were stimulated with mouse HER2 peptide pools, followed by intracellular cytokine staining (ICCS) and FCM to quantify proportions of functional T cells (Fig. S4A). The FCM data showed that 40 μg FNP-adjuvanted mHER2_aHPF nanoparticle vaccines were unable to upregulate TNF-α^+^CD4^+^ T cells, while both 60 μg and 80 μg FNP-adjuvanted nanoparticle vaccines significantly increased the percentages of TNF-α^+^CD4^+^ T cells when compared to unadjuvanted vaccines (Fig. 3C). The percentages of IFNγ^+^CD4^+^ T cells were also significantly upregulated by the vaccines adjuvanted with 60 μg and 80 μg FNP (Fig. 3D). However, when compared to the vaccines adjuvanted with 60 μg FNP, those adjuvanted with 80 μg FNP resulted in a significant decrease in IFNγ^+^CD4^+^ T cells. Increasing the FNP adjuvant dose also increased the proportions of TNF-α^+^CD8^+^ T cells (Fig. 3E). The percentages of IFNγ^+^CD8^+^ T cells and Granzyme B^+^CD8^+^ T cells reached their peaks in 60 μg FNP-adjuvanted vaccines, although these cells were all significantly increased in vaccines adjuvanted with both 60 μg and 80 μg FNP (Fig. 3F and G). Furthermore, we assessed the humoral immune responses elicited by these vaccines via measuring mHER2-specific antibodies. The ELISA results showed that nanoparticle vaccines adjuvanted with 60 μg and 80 μg FNP significantly upregulated mHER2-specific IgG antibodies in comparison with unadjuvanted vaccines (Fig. 3H and S4B). These results demonstrated that FNP acted as a potent immunoadjuvant that significantly enhanced both cellular and humoral immune responses elicited by the mHER2_aHPF nanoparticle vaccine. Given the optimal immune responses elicited by vaccines adjuvanted with 60 μg FNP, the subsequent vaccination experiments employed 60 μg FNP as an additional adjuvant.

The CpG oligodeoxynucleotides (CpG ODNs) have been extensively utilized as potent adjuvants in tumor vaccines by activating the Toll-like receptor 9 (TLR9) signaling pathway in APCs to promote type I interferon and proinflammatory cytokine production [51]. In contrast, FNP has been reported to engage multiple TLRs and to signal through both MyD88-dependent and TRIF-dependent pathways, thereby driving DC maturation and Th1-biased immune responses [57,58]. Given that CpG and FNP engage distinct yet complementary innate sensing mechanisms, we profiled the innate signaling pathways engaged by each adjuvant alone and in combination. DC2.4 cells were treated with PBS, CpG, FNP, or the CpG-FNP combination. The expression of TLRs and downstream effector cytokines was quantified by quantitative real-time PCR (RT-qPCR) (Table S1). Our results showed that FNP alone significantly upregulated TLR1 and TLR3, whereas CpG selectively activated TLR9 (Fig. 3I–Q). Notably, the CpG-FNP combination induced the broadest TLR activation, significantly elevating TLR1, TLR2, TLR3, TLR4, TLR8, and TLR9 relative to the unadjuvanted control. TLR4, which uniquely couples to both the MyD88 and TRIF adaptor pathways, was significantly upregulated only by the combination, suggesting that CpG and FNP provide convergent signals at this receptor (Fig. 3L). In contrast, the expression of the NLRP3 inflammasome component remained unchanged across all treatments, indicating that FNP drives innate activation primarily through TLR signaling rather than inflammasome assembly, consistent with previous reports distinguishing C60(OH)x from gadolinium-containing metallofullerenols (Fig. 3R) [59]. Consistent with this broad TLR engagement, the CpG-FNP combination markedly amplified downstream effector cytokine transcription. The NF-κB-driven cytokines IL-1β, IL-6, and IL-12b, as well as the TRIF-IRF3 hallmark cytokine IFN-β1, were synergistically upregulated by the dual-adjuvant combination relative to either agent alone (Fig. 3S–V). These results demonstrated that CpG and FNP activated complementary TLR pathways that converged on NF-κB and IRF3 to amplify innate immune activation. Based on this synergy, the CpG-FNP dual-adjuvant system was employed in all subsequent immunization experiments.

### 3.4 HER2 nanoparticle vaccines demonstrate enhanced lymphatic targeting and immunogenicity

**Fig. 4:**
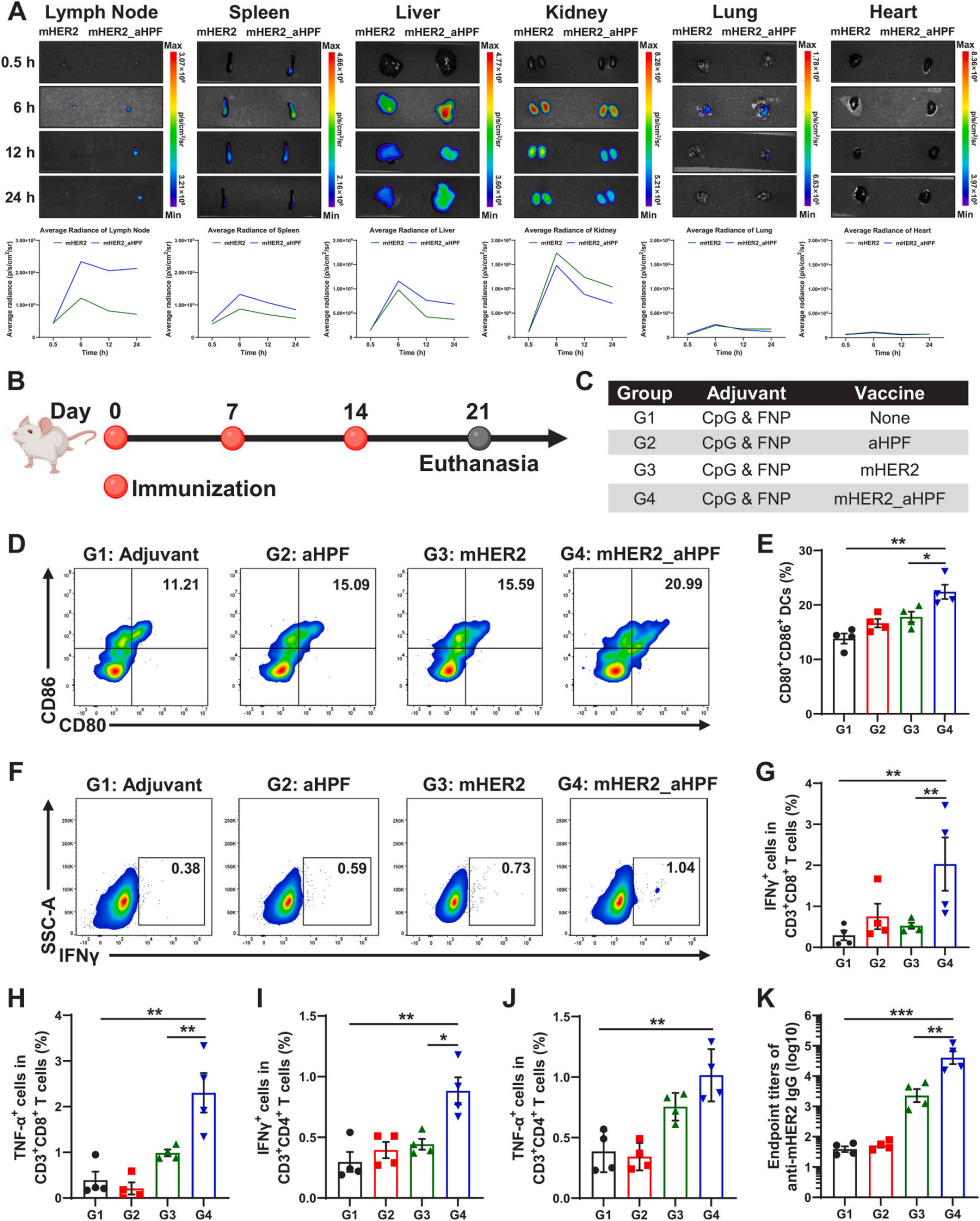
**mHER2 nanoparticle vaccines demonstrate enhanced lymphatic targeting and immune responses.** (A) Cy7-labeled mHER2 and mHER2_aHPF (0.4 nmol per mouse) were injected subcutaneously in the cervicodorsal region of female BALB/c mice. The lymph node, spleen, liver, kidney, lung, and heart were excised at 0.5, 6, 12, and 24 h post-injection and imaged *ex vivo*. Absolute average radiance (p/s/cm^2^/sr) was quantified at each time point. (B) Schematic representation of vaccination timeline in female BALB/c mice (*n* = 4 per group) using adjuvanted mouse HER2 vaccines. Each mouse received vaccinations on Day 0 (prime), 7 (boost), and 14 (booster), followed by euthanasia on Day 21 to harvest immune cells and sera for the quantification of immune responses. (C) Vaccination study groups. All four groups were adjuvanted with 30 μg CpG and 60 μg FNP. G1: treatment with adjuvant only. G2: equal moles of aHPF. G3: equal moles of mHER2. G4: equal moles of mHER2_aHPF. (D-E) DCs within inguinal lymph nodes of each mouse were analyzed by FCM (D), and percentages of mature DCs were determined by gating CD80^+^CD86^+^ DCs and normalized to total numbers of DCs (E). (F-H) Splenocytes were stimulated with mouse HER2 peptide pools and subjected to ICCS. Representative flow cytometry plots of IFNγ^+^ CD8^+^ T cells were shown in (F). The percentages of IFNγ^+^ (G) and TNF-α^+^ (H) cells among CD3^+^CD8^+^ T cells were quantified. (I-J) Percentages of antigen-specific IFNγ^+^ (I) and TNF-α^+^ (J) CD4^+^ T cells were measured and quantified as in (G-H). (K) Endpoint titers of mouse HER2-specific IgG within sera were quantified by ELISA. Data in (E) and (G-K) were presented as mean ± SEM. *P* values were calculated by one-way ANOVA with Tukey's multiple comparisons tests. *P < 0.05, **P < 0.01, ***P < 0.001.

Our previous results have demonstrated that HER2 nanoparticle vaccines promoted antigen uptake and DC maturation. We next examined whether the nanoparticle format enhanced lymphatic targeting *in vivo*. Cy7-labeled mHER2 and mHER2_aHPF were injected subcutaneously in the cervicodorsal region of female BALB/c mice. The lymph node, spleen, liver, kidney, lung, and heart were excised at 0.5, 6, 12, and 24 h post-injection for *ex vivo* fluorescence imaging. Both mHER2 and mHER2_aHPF distributed primarily to the liver and kidney, with signal intensity peaking at 6 h and gradually declining thereafter (Fig. 4A). The monomeric mHER2 signal decayed more rapidly than that of mHER2_aHPF, with higher accumulation in the kidney, indicating that monomeric HER2 was more rapidly eliminated via the renal route. In contrast, mHER2_aHPF accumulated preferentially in the lymph node and spleen relative to the monomer. At 24 h post-injection, mHER2_aHPF exhibited 11.35% of the total organ signal in the lymph node and 4.57% in the spleen, compared with 4.00% and 3.28% for mHER2, respectively (Fig. S5A). These results demonstrated that the nanoparticle format significantly enhanced lymphatic drainage and lymph node retention of HER2 antigens. The sustained lymph node retention of mHER2_aHPF at 24 h post-injection provided an extended window for antigen uptake by lymph node-resident DCs.

After confirming the enhanced lymphatic targeting of nanoparticle vaccines, we subsequently evaluated their capacity to elicit antigen-specific immune responses. All female BALB/c mice received three doses of CpG-FNP-adjuvanted vaccines on Day 0, 7, and 14 (Fig. 4B). Mice in Group 1 (G1) were administered with the adjuvant only, whereas mice in G2, G3, and G4 received equal moles of aHPF, mHER2, and mHER2_aHPF, respectively (Fig. 4C). One week after the third vaccination, mice were euthanized to harvest blood samples, lymph nodes, and spleens. The FCM analysis of DCs in inguinal lymph nodes revealed that mHER2_aHPF vaccination activated higher proportions of CD80^+^CD86^+^ mature DCs compared to mHER2 monomeric vaccines (Fig. 4D and E, and S5B). This enhanced DC maturation, together with the prolonged lymph node retention observed in the biodistribution analysis, suggested that the nanoparticle-mediated sustained antigen delivery contributed to more effective priming of lymph node-resident DCs over the course of repeated immunization. Furthermore, mHER2_aHPF significantly increased the percentages of IFNγ^+^ and TNF-α^+^ CD8^+^ T cells and CD4^+^ T cells in the spleens relative to mHER2 (Fig. 4F–J). Sera from mHER2_aHPF-vaccinated mice exhibited higher levels of mHER2-specific IgG titers compared to those from monomer-vaccinated mice (Fig. 4K and S5C). These antibodies mediated potent antibody-dependent cellular phagocytosis (ADCP) of mHER2-expressing 4T1 cells by RAW264.7 macrophages (Fig. S5D–S5F). To confirm that the observed phagocytosis was Fc-mediated, RAW264.7 macrophages were pre-incubated with anti-mouse CD16/CD32 antibody to block Fcγ receptors. Fc receptor blockade markedly reduced phagocytosis in the mHER2_aHPF group, confirming that the enhanced ADCP activity required Fcγ receptor engagement (Fig. S5G and S5H).

**Fig. 5:**
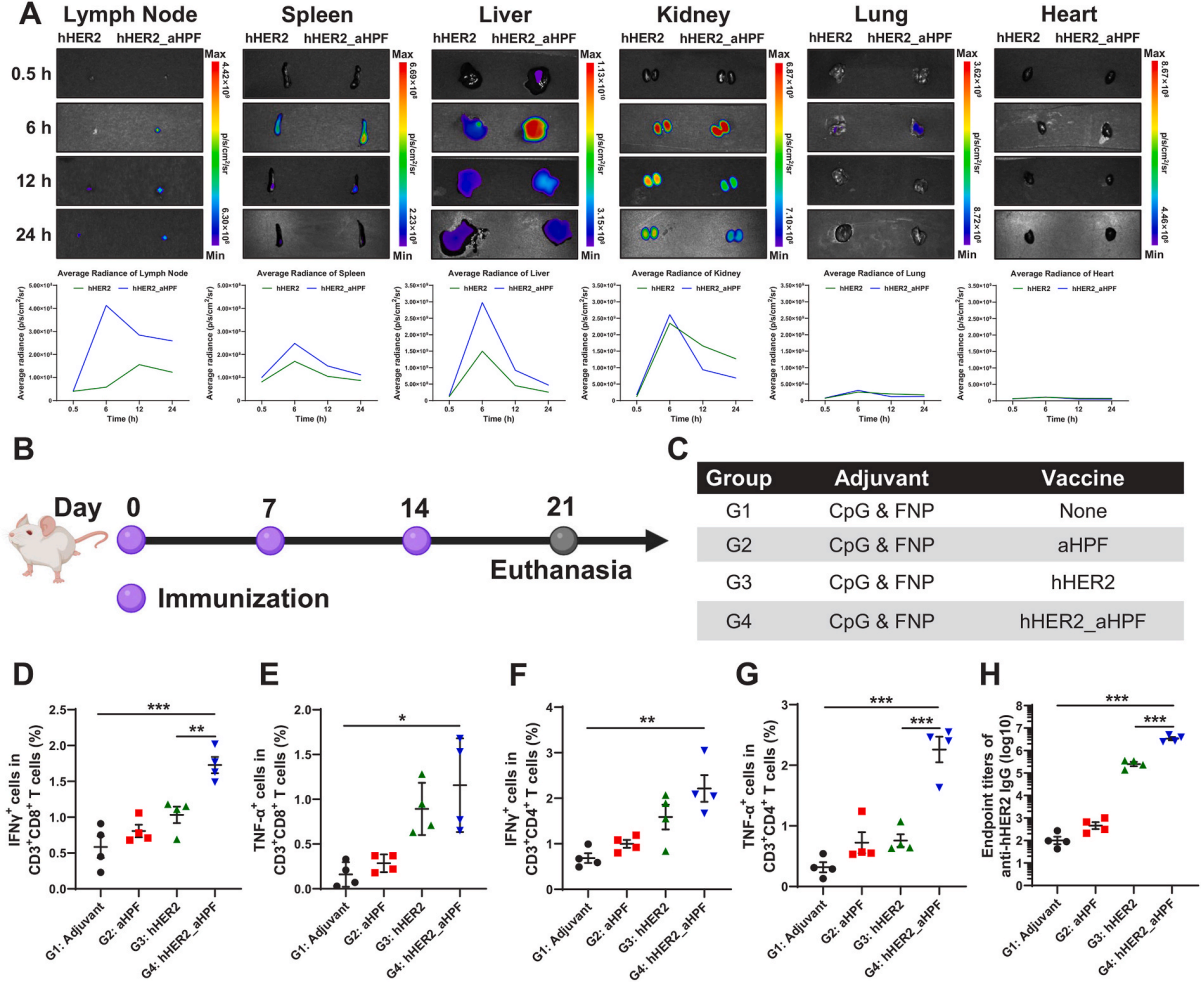
**Nanoparticle-mediated lymphatic drainage enhances hHER2-specific immune responses.** (A) Female BALB/c mice received subcutaneous injections of Cy7-labeled hHER2 and hHER2_aHPF (0.4 nmol per mouse) in the cervicodorsal region. At 0.5, 6, 12, and 24 h post-injection, the lymph node, spleen, liver, kidney, lung, and heart were collected. *Ex vivo* fluorescence images were acquired, and average radiance (p/s/cm^2^/sr) was quantified for each organ at each time point. (B-C) The vaccination timeline and experimental groups (*n* = 4 per group) were set as in Fig. 4B–C, except that antigens used in this study were derived from human HER2. G1: adjuvant only. G2: aHPF. G3: hHER2. G4: hHER2_aHPF. (D-G) Percentages of IFNγ^+^CD8^+^ (D), TNF-α^+^CD8^+^ (E), IFNγ^+^CD4^+^ (F), and TNF-α^+^CD4^+^ (G) T cells were quantified as in Fig. 4G–J. HER2 peptide pools used in this study were derived from human HER2. (H) Endpoint titers of human HER2-specific IgG were quantified by ELISA. Data in (D-H) were presented as mean ± SEM. *P* values were calculated by one-way ANOVA with Tukey's multiple comparisons tests. *P < 0.05, **P < 0.01, ***P < 0.001.

We next assessed whether the hHER2_aHPF nanoparticle vaccine exhibited the same lymphatic targeting and immune activation profile as its murine counterpart. Cy7-labeled hHER2 and hHER2_aHPF were injected subcutaneously in the cervicodorsal region. Major organs were collected at 0.5, 6, 12, and 24 h for *ex vivo* imaging. Consistent with the mHER2 results, hHER2_aHPF accumulated preferentially in the lymph node and spleen, whereas the monomeric hHER2 was cleared more rapidly and showed greater renal retention (Fig. 5A). At 24 h, hHER2_aHPF distributed 15.22% and 6.58% of total organ signal to the lymph node and spleen, compared with 6.21% and 4.40% for hHER2 (Fig. S6A). Lung and heart signals remained low for both formulations. For immune response evaluation, the vaccination schedule and group assignments followed the same design as the mHER2 experiment, with hHER2 and hHER2_aHPF replacing the murine antigens in G3 and G4 (Fig. 5B and C). Immunization with hHER2_aHPF elicited robust polyfunctional T-cell responses, with elevated frequencies of IFNγ^+^ and TNF-α^+^ cells among both CD8^+^ and CD4^+^ T-cell compartments relative to monomeric hHER2 (Fig. 5D–G). Anti-hHER2 IgG titers were also significantly higher in the nanoparticle vaccine group, and these antibodies drove efficient ADCP of hHER2-expressing 4T1 cells by RAW264.7 macrophages (Fig. 5H and S6B-S6D). Pre-incubation of RAW264.7 cells with anti-mouse CD16/CD32 antibody substantially diminished phagocytosis, establishing that the ADCP activity of hHER2_aHPF immune sera was likewise dependent on Fcγ receptor engagement (Fig. S6E and S6F).

To confirm that the robust immunogenicity of these vaccines was not accompanied by systemic toxicity, a comprehensive safety evaluation was performed in mice receiving three doses of the indicated formulations. Hematoxylin and eosin (H&E) staining of the myocardium, liver, spleen, lung, and kidney revealed no pathological changes across the Mock, Adjuvant, mHER2_aHPF, and hHER2_aHPF groups (Fig. S7A). Hematological parameters remained within normal reference ranges for all groups, including red blood cell (RBC), platelet (PLT), hemoglobin (HGB), and hematocrit (HCT) (Fig. S7B–S7E). Serum biochemical indicators of hepatic and renal function were also unaffected, including alanine aminotransferase (ALT), aspartate aminotransferase (AST), alkaline phosphatase (ALP), total bilirubin (TBIL), γ-glutamyl transferase (γ-GT), total bile acids (TBA), urea (UREA), and creatinine (CREA) (Fig. S7F–S7M). Serum IL-6 and TNF-α levels in the mHER2_aHPF and hHER2_aHPF groups were significantly elevated relative to the Mock control (Fig. S7N and S7O). This elevation was consistent with productive vaccine-induced innate immune activation and remained far below the concentrations associated with cytokine release syndrome [[60], [61], [62]]. Collectively, these results demonstrated that both mHER2 and hHER2 nanoparticle vaccines effectively targeted the lymphatic system, induced potent cellular and humoral immune responses, and maintained a favorable *in vivo* safety profile.

### 3.5 HER2 nanoparticle vaccines inhibit HER2-positive tumor growth

**Fig. 6:**
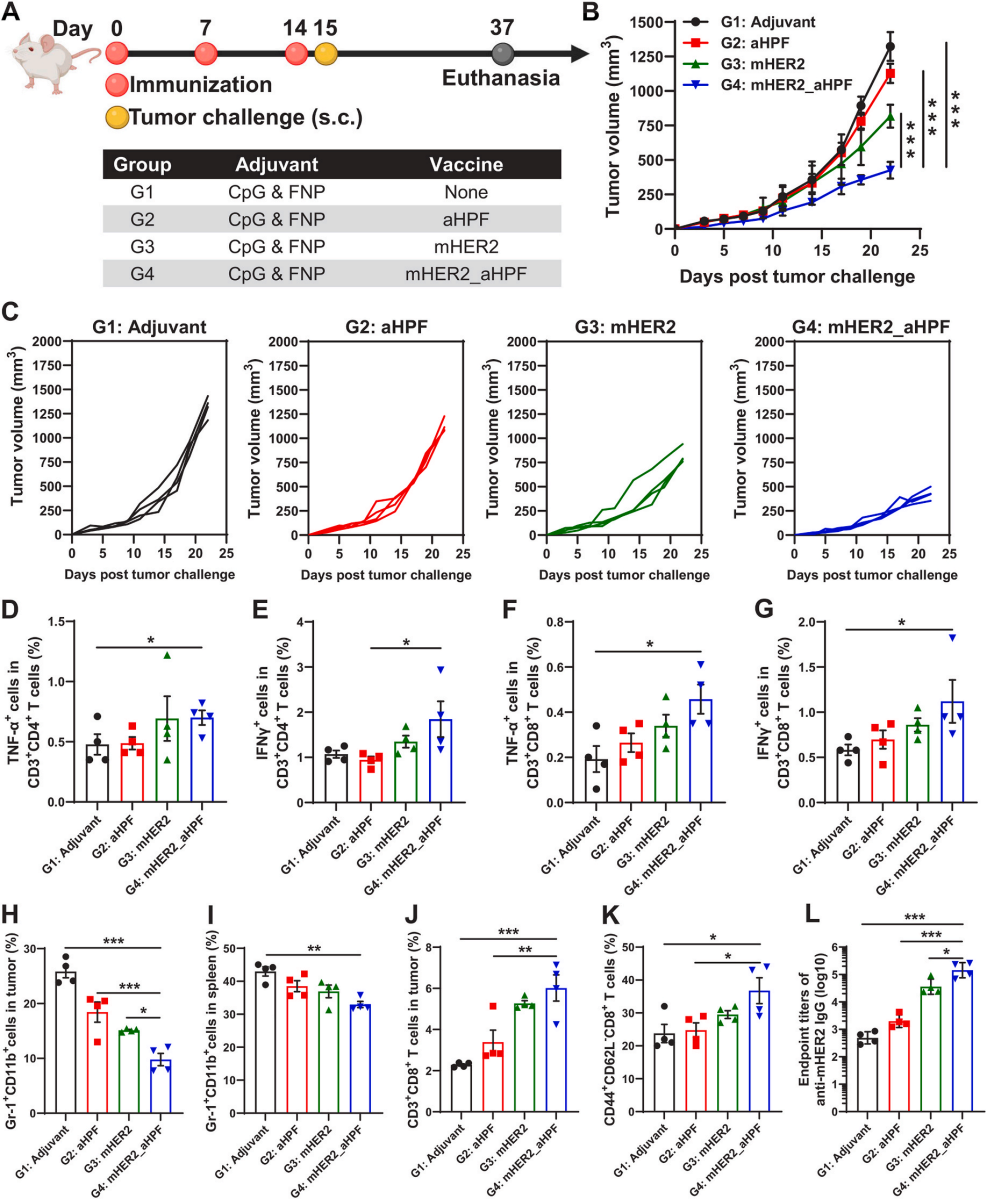
**mHER2 nanoparticle vaccines inhibit the growth of mHER2-positive tumors.** (A) Schematic of prophylactic vaccination with mHER2 nanoparticle vaccines (*n* = 4 per group). Each female BALB/c mouse received three doses of vaccines on Day 0, 7, and 14, followed by subcutaneous (s.c.) tumor challenge using 4T1-mHER2-Luc cells. Mice were euthanized on Day 22 post-tumor challenge. Mice in G1 received CpG-FNP adjuvant only, while mice in G2, G3, and G4 received equal moles of aHPF, mHER2, mHER2_aHPF, respectively. (B) Tumor dimensions of each mouse were measured every two days post tumor challenge to determine tumor volumes. (C) The tumor growth curve of each mouse in each group was tracked and presented as a separate trajectory. (D-G) Splenocytes isolated from each mouse were stimulated with mouse HER2 peptides, followed by ICCS and FCM to quantify percentages of TNF-α^+^CD4^+^ (D), IFNγ^+^CD4^+^ (E), TNF-α^+^CD8^+^ (F), and IFNγ^+^CD8^+^ (G) T cells. (H-I) Percentages of myeloid-derived suppressor cells (MDSCs) within tumors and spleens were analyzed by gating CD45^+^/CD11b^+^/Gr-1^+^ cells and normalized to total numbers of CD45^+^/CD11b^+^ cells. (J) Percentages of tumor-infiltrating CD3^+^CD8^+^ T cells were quantified by FCM and normalized to total cells. (K) Percentages of splenic CD3^+^/CD8^+^/CD44^+^/CD62L^−^ effector-memory T (T_EM_) cells were quantified by normalizing to total numbers of CD8^+^ T cells. (L) Endpoint titers of mHER2-specific IgG within sera were quantified by ELISA. Data in (B) and (D-L) were presented as mean ± SEM. *P* values were calculated by two-way ANOVA (B) or one-way ANOVA (D-L) with Dunnett's multiple comparisons tests. *P* values in (B) were derived from comparisons conducted on Day 22. *P < 0.05, **P < 0.01, ***P < 0.001.

To evaluate the prophylactic efficacy of HER2 nanoparticle vaccines *in vivo*, female BALB/c mice were subcutaneously (s.c.) challenged with mHER2-expressing 4T1 cells after three doses of vaccination (Fig. 6A). The tumor volumes in each mouse were monitored for 22 days, followed by euthanasia when the tumor volume in mice from the control group reached 1000 mm^3^. The results showed that mice in the adjuvant group (G1) and the aHPF nanoparticle core group (G2) exhibited high tumor growth rates, reaching 1000 to 1250 mm^3^ at 19 days post-tumor challenge (Fig. 6B). In contrast, the administration of both mHER2 and mHER2_aHPF vaccines significantly suppressed tumor progression. The prophylactic efficacy of mHER2_aHPF vaccines was higher than that of monomeric mHER2 vaccines. On Day 22 after tumor challenge, the tumor volumes of mHER2 and mHER2_aHPF were less than 1000 and 500 mm^3^, respectively. The individual tumor growth curves of each mouse confirmed that mHER2_aHPF nanoparticle vaccines effectively delayed tumor progression, highlighting their potential for tumor prevention (Fig. 6C).

To elucidate the immunological mechanisms underlying the tumor suppression induced by mHER2 nanoparticle vaccines, splenocytes from euthanized mice were subjected to functional T-cell quantifications. The FCM data revealed that the administration of mHER2_aHPF nanoparticle vaccines induced higher percentages of mHER2-specific TNF-α^+^ and IFNγ^+^ CD4^+^ T cells when compared with the other groups (Fig. 6D and E). Both TNF-α^+^ and IFNγ^+^ CD8^+^ T cells were also significantly elevated upon mHER2_aHPF vaccination, demonstrating enhanced cytotoxic T lymphocyte (CTL) responses (Fig. 6F and G). One of the significant obstacles to intrinsic anti-tumor immunity is the expansion of myeloid-derived suppressor cells (MDSCs) in tumors and spleens, which contributes to the establishment of an immunosuppressive tumor microenvironment by suppressing T-cell immune responses [63]. Consistent with the established immunosuppressive features of 4T1 tumors, the challenge of mHER2-positive tumors notably increased the proportions of Gr-1^+^CD11b^+^ MDSCs in tumors and spleens (Fig. 6H and I and S8A). Conversely, the prophylactic vaccination with mHER2_aHPF nanoparticle vaccines significantly inhibited the expansion of MDSCs compared to the other groups. Consequently, mHER2_aHPF vaccination also significantly upregulated the percentages of tumor-infiltrating CD8^+^ T cells within tumors, achieving effective anti-tumor immunity (Fig. 6J). Moreover, the administration of mHER2_aHPF increased the proportions of CD44^+^CD62L^−^CD8^+^ effector-memory T (T_EM_) cells in spleens, demonstrating enhanced immunological memory following vaccination (Fig. 6K and S8B). Concurrently, mHER2_aHPF nanoparticle vaccines elicited higher titers of mHER2-specific antibodies in sera compared to the other groups, which was consistent with our previous observations (Fig. 6L and S8C). In a separate cohort of mice subjected to the same prophylactic immunization and tumor challenge schedule, tumor tissues from the Adjuvant and mHER2_aHPF groups were harvested on Day 37 and analyzed for mHER2 expression by flow cytometry (Fig. S8D). The percentage of mHER2-positive tumor cells in the mHER2_aHPF group was comparable to that in the Adjuvant control and 4T1-mHER2 cells, with no evidence of antigen downregulation or the emergence of HER2-negative populations (Fig. S8E). Taken together, our above results demonstrated that prophylactic vaccination with mHER2_aHPF nanoparticle vaccines effectively inhibited tumor progression by inducing potent cellular and humoral immune responses and suppressing MDSC expansion.

### 3.6 HER2 nanoparticle vaccines synergize with ICB therapies to inhibit pulmonary metastasis of HER2-positive tumors

**Fig. 7:**
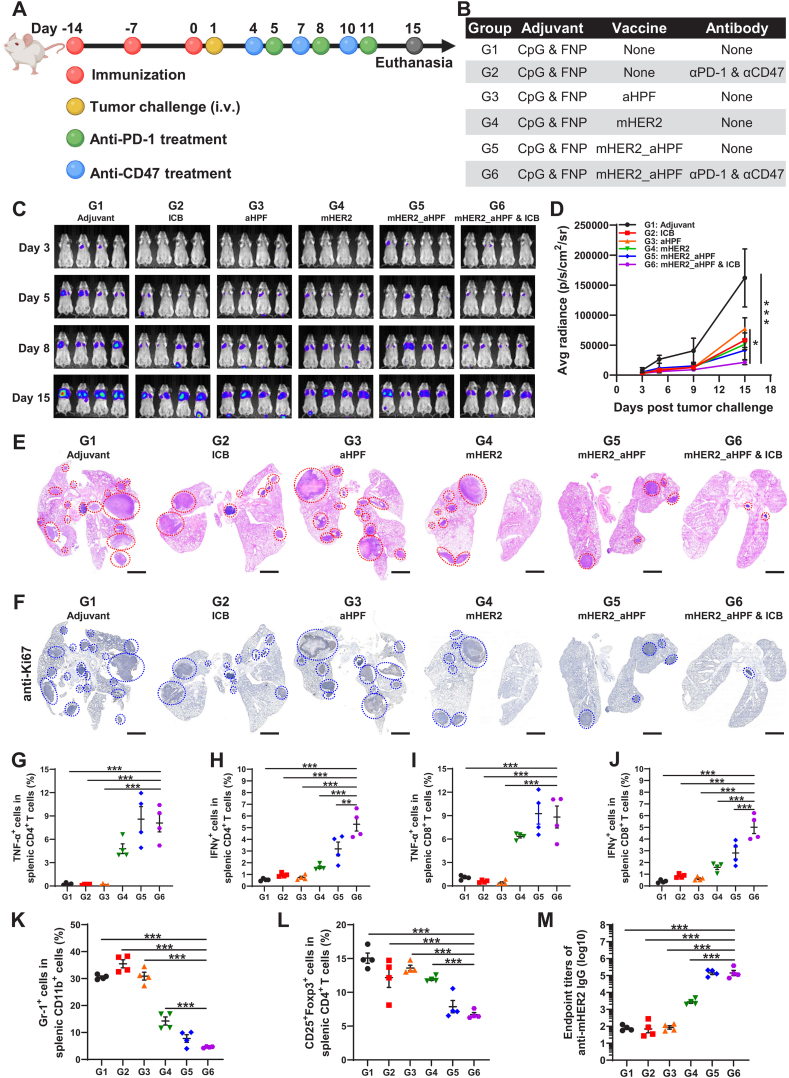
**Prophylactic mHER2 nanoparticle vaccination synergizes with ICB therapies to inhibit tumor progression.** (A) Schematic of prophylactic vaccination combined with immune checkpoint blockade (ICB) therapies (*n* = 4 per group). Each female BALB/c mouse received three doses of vaccines on Day −14, −7, and 0, followed by intravenous (i.v.) tumor challenge using 4T1-mHER2-Luc cells on Day 1. Intraperitoneal anti-CD47 (αCD47) and αPD-1 ICB therapies were alternately administered on Day 4, 5, 7, 8, 10, and 11. All mice were euthanized on Day 15. (B) Groups of vaccination and ICB therapies. G1: adjuvant only. G2: αPD-1 and αCD47 only. G3: aHPF. G4: mHER2. G5: mHER2_aHPF. G6: mHER2_aHPF combined with αPD-1 and αCD47. (C) Bioluminescence imaging of each mouse was conducted on Day 3, 5, 8, and 15 to monitor lung metastasis. (D) Statistical quantifications of radiances measured by bioluminescence imaging at each time point. (E) Representative hematoxylin and eosin (H&E) staining images of mouse lungs from each group. The densely eosinophilic (pink) regions, circled in red, corresponded to lung metastatic nodules. Scale bar, 2 mm. (F) Representative immunohistochemical (IHC) images of mouse lungs from each group were obtained using antibodies against Ki67. Scale bar, 2 mm. (G-J) Splenocytes obtained from each mouse were stimulated with mouse HER2 peptides, followed by ICCS and FCM to quantify percentages of TNF-α^+^ (G) and IFNγ^+^ (H) splenic CD4^+^ T cells, as well as TNF-α^+^ (I) and IFNγ^+^ (J) splenic CD8^+^ T cells. (K) Percentages of MDSCs within spleens were quantified by gating Gr-1^+^ splenic CD11b^+^ cells. (L) Percentages of CD45^+^/CD3^+^/CD4^+^/CD25^+^/Foxp3^+^ regulatory T cells (Tregs) were analyzed by normalizing to total numbers of splenic CD4^+^ T cells. (M) Endpoint titers of mHER2-specific IgG within sera were quantified by ELISA. Data in (D) and (G-M) were presented as mean ± SEM. *P* values were calculated by two-way ANOVA (D) or one-way ANOVA (G-M) with Dunnett's multiple comparisons tests. *P* values in (D) were derived from comparisons conducted on Day 15. *P < 0.05, **P < 0.01, ***P < 0.001.

Two additional barriers to anti-tumor immunity are the upregulation of PD-1 on activated T cells and the upregulation of CD47 on tumor cells during chronic stimulation. PD-L1 is widely expressed on tumor cells and interacts with PD-1 on T cells to inhibit T-cell proliferation and reduce cytokine production, thereby contributing to the formation of exhausted T (T_EX_) cells [64]. Meanwhile, CD47 on tumor cells binds to SIRPα on macrophages to suppress the phagocytic activity of macrophages, effectively sending a “don't eat me” signal [65]. Monoclonal antibody (mAb) therapies targeting both PD-1 and CD47 immune checkpoint axes have been shown to restore innate-adaptive crosstalk and reshape the anti-tumor microenvironment [66,67]. Previously, we have demonstrated that FNP promotes the exposure of the calreticulin on tumor cells, thereby providing the “eat me” signal to macrophages for phagocytosis [56]. Therefore, we hypothesized that the CpG-FNP dual-adjuvanted HER2 nanoparticle vaccines could synergize with anti-PD-1 and anti-CD47 immune checkpoint blockade (ICB) therapies to effectively suppress tumor progression. Mice were prophylactically immunized with mHER2_aHPF as described in Fig. 6. One day post-immunization, 4T1 cells expressing both mouse HER2 and luciferase were intravenously (i.v.) injected to female BALB/c mice to establish lung metastatic models (Fig. 7A). The anti-PD-1 and anti-CD47 ICB therapies were alternately administered to mice in the adjuvant-only group (G2) and the mHER2_aHPF-vaccinated group (G6) on Day 4, 5, 7, 8, 10, and 11 (Fig. 7B). Lung metastases of mouse HER2-positive tumors were monitored via *in vivo* bioluminescence imaging. The results demonstrated that mice in the adjuvant-only group rapidly developed lung metastases, as evidenced by the continuous increase in bioluminescent signals, which indicated ongoing metastatic progression (Fig. 7C). Both ICB therapies and mHER2 antigen vaccinations significantly delayed the development of lung metastases compared to the adjuvant-only group (Fig. 7D). Particularly, the combination of mHER2_aHPF vaccination and ICB therapies demonstrated the highest efficacy in inhibiting tumor progression. The H&E staining results indicated that mouse lungs of the adjuvant and aHPF groups were scattered with metastatic nodules (Fig. 7E). In contrast, both the ICB therapy-only and mHER2 vaccination-only groups exhibited reduced numbers of metastatic nodules. Specifically, only a few of these nodules were visible in mouse lungs of the mHER2_aHPF and ICB co-treatment group (Fig. 7E). Consistently, the combination treatment group also exhibited the lowest Ki67 positivity (Fig. 7F). These results demonstrated that prophylactic vaccination with mHER2_aHPF synergized with ICB therapies to effectively inhibit the lung metastases of mouse HER2-positive tumors.

To elucidate the fundamental mechanisms underlying the enhanced anti-tumor efficacy of mHER2_aHPF and ICB co-treatment, splenocytes and sera of each mouse were isolated for the quantifications of immune responses. Both the mHER2_aHPF-only and the combined treatment groups exhibited higher percentages of polyfunctional T cells in spleens compared to the other groups, as demonstrated by elevated percentages of TNF-α^+^ and IFNγ^+^ CD4^+^ T cells, as well as TNF-α^+^ and IFNγ^+^ CD8^+^ T cells (Fig. 7G–J). The combination of ICB therapies with mHER2_aHPF exhibited higher proportions of IFNγ^+^CD4^+^ T cells and IFNγ^+^CD8^+^ T cells compared to mHER2_aHPF vaccination only (Fig. 7H and J). Furthermore, the combined treatment significantly inhibited the expansion of MDSCs, as evidenced by the lowest percentages of Gr-1^+^CD11b^+^ MDSCs in spleens (Fig. 7K). The elevated levels of regulatory T cells (Tregs) have been shown to suppress the activities of CTLs and APCs during tumor progression [68]. Our results demonstrated that the prophylactic vaccination with mHER2_aHPF, as well as its combination with ICB therapies, significantly inhibited Treg expansion, as demonstrated by lower percentages of CD25^+^/Foxp3^+^ splenic CD4^+^ T cells compared to the other groups (Fig. 7L and S9A). Consistent with our previous observations, mHER2_aHPF nanoparticle vaccines also elicited higher mHER2-specific IgG titers in sera compared to the other groups, which could contribute to enhanced ADCP of mHER2-expressing 4T1 cells (Fig. 7M and S9B). Altogether, the prophylactic mHER2_aHPF vaccination synergized with ICB therapies to effectively activate potent cellular and humoral immune responses, inhibit the expansion of immunosuppressive MDSCs and Tregs, and consequently impede the progression of lung metastases.

### 3.7 In vivo therapeutic efficacy of mouse HER2 nanoparticle vaccines

**Fig. 8:**
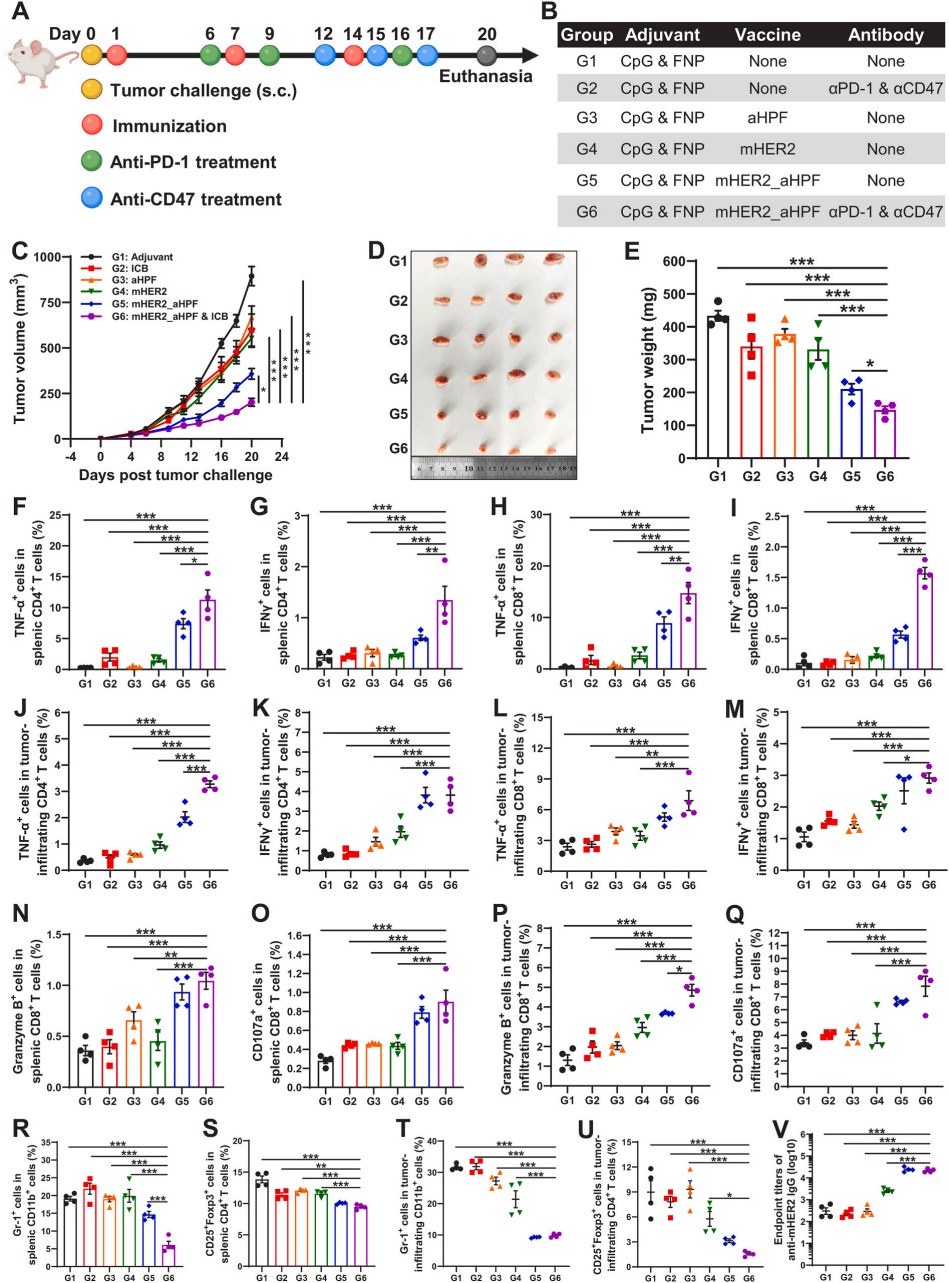
**Therapeutic mHER2 nanoparticle vaccination synergizes with ICB therapies to enhance tumor suppression.** (A) Schematic of therapeutic vaccination combined with ICB therapies (*n* = 4 per group). Female BALB/c mice were subjected to subcutaneous (s.c.) tumor challenge using 4T1-mHER2-Luc cells. Three doses of vaccines were administered on Day 1, 7, and 14, while αPD-1 and αCD47 ICB therapies were alternately administered on Day 6, 9, 12, 15, 16, and 17. All mice were euthanized on Day 20. (B) Vaccination and ICB therapy groups. G1: adjuvant only. G2: αPD-1 and αCD47 only. G3: aHPF. G4: mHER2. G5: mHER2_aHPF. G6: mHER2_aHPF combined with αPD-1 and αCD47. (C) Tumor volumes were quantified every two or three days by measuring tumor dimensions. (D) Representative tumor images of each mouse in each group were shown. Tumors were isolated on Day 20 post-challenge. (E) Weights of isolated tumors were quantified for each group. (F-I) Percentages of TNF-α^+^ (F) and IFNγ^+^ (G) splenic CD4^+^ T cells, as well as TNF-α^+^ (H) and IFNγ^+^ (I) splenic CD8^+^ T cells, were quantified using splenocytes stimulated by mouse HER2 peptides. (J-M) Tumor-infiltrating lymphocytes were stimulated with mouse HER2 peptides, followed by ICCS and FCM to quantify percentages of TNF-α^+^ (J) and IFNγ^+^ (K) CD4^+^ T cells, as well as TNF-α^+^ (L) and IFNγ^+^ (M) CD8^+^ T cells. (N-Q) Percentages of Granzyme B^+^ and CD107a^+^ CD8^+^ T cells within spleens (N-O) and tumors (P-Q) were respectively quantified by FCM. (R) Percentages of MDSCs within spleens were quantified by gating Gr-1^+^CD11b^+^ cells and then normalized to total numbers of splenic CD11b^+^ cells. (S) Percentages of CD45^+^/CD3^+^/CD4^+^/CD25^+^/Foxp3^+^ Tregs were analyzed by normalizing to total numbers of splenic CD4^+^ T cells. (T-U) Tumor-infiltrating MDSCs (T) and Tregs (U) were quantified as in (R-S). (V) Endpoint titers of mHER2-specific IgG in sera from each group were quantified by ELISA. Data in (C) and (E-V) were presented as mean ± SEM. *P* values were calculated by two-way ANOVA (C) or one-way ANOVA (E-V) with Dunnett's multiple comparisons tests. *P* values in (C) were derived from comparisons conducted on Day 20. *P < 0.05, **P < 0.01, ***P < 0.001.

After confirming the enhanced prophylactic efficacy of nanoparticle vaccines, we then investigated the therapeutic potential of mHER2 nanoparticle vaccines to suppress tumor progression *in vivo*. All female BALB/c mice were subcutaneously (s.c.) inoculated with mHER2-expressing 4T1 cells, followed by vaccination with three doses of vaccines on Day 1, 7, and 14 post-inoculation (Fig. 8A). Additionally, the anti-PD-1 and anti-CD47 ICB therapies were alternately administered on Day 6, 9, 12, 15, 16, and 17. Mice in G1 group received CpG-FNP dual-adjuvant only, while mice in G2, G3, G4, and G5 were co-administered with ICB antibodies, aHPF, mHER2, and mHER2_aHPF, respectively (Fig. 8B). Mice in G6 were administered the combined treatment of mHER2_aHPF and ICB therapies. All mice were euthanized on Day 20 when the tumor volume reached 1000 mm^3^. The results indicated that mice in the adjuvant (G1) group displayed high tumor growth rates (Fig. 8C). Mice in the ICB therapy (G2), aHPF (G3), monomeric mHER2 (G4) groups also showed limited anti-tumor efficacy. In contrast, both mHER2_aHPF vaccination and its combination with ICB therapies notably inhibited tumor progression. Among all groups, the combined treatment showed the highest efficacy in tumor suppression. The combined treatment of mHER2_aHPF and ICB therapies led to a smaller tumor morphology and lower tumor weight compared to the other groups (Fig. 8D and E). These results demonstrated that mHER2 nanoparticle vaccines exhibited potent therapeutic efficacy against HER2-positive tumors and synergized with ICB therapies to achieve the enhanced tumor suppression.

To investigate the underlying immune mechanisms responsible for the enhanced therapeutic potency of mHER2 nanoparticle vaccines, both the spleen and tumor tissues of euthanized mice were harvested for the quantification of immune responses. Splenocytes and tumor-infiltrating lymphocytes were stimulated with mouse HER2 peptides, followed by FCM analysis to assess antigen-specific T-cell responses. The results demonstrated that the proportions of polyfunctional T cells were significantly upregulated following mHER2_aHPF vaccination, as evidenced by the increased percentages of TNF-α^+^ and IFNγ^+^ CD4^+^ T cells, along with TNF-α^+^ and IFNγ^+^ CD8^+^ T cells (Fig. 8F–I). The combined treatment of mHER2_aHPF vaccination and ICB therapies induced significantly higher percentages of these cells compared to the other groups. Similarly, mHER2_aHPF vaccination and its combination with ICB therapies also elicited potent polyfunctional T-cell immune responses in tumors, resulting in elevated levels of TNF-α^+^ and IFNγ^+^ tumor-infiltrating T cells (Fig. 8J–M). These results indicated that mHER2_aHPF nanoparticle vaccines, along with their combination with ICB therapies, established potent anti-tumor immunity in both lymphoid organs and tumors. The antigen-specific CTLs suppress target cells via both non-lytic and lytic mechanisms [69]. The non-lytic mechanism is manifested by the elevated levels of TNF-α and IFNγ secreted by activated CD8^+^ T cells, while these CD8^+^ T cells also employ the lytic mechanism by upregulating Granzyme B along with perforin to directly trigger the apoptosis of target tumor cells. Additionally, CD8^+^ T cells also upregulate CD107a to promote degranulation and the concomitant release of Granzyme B from lytic granules [70]. Consistently, mHER2_aHPF vaccination and its combination with ICB therapies significantly upregulated both splenic and tumor-infiltrating Granzyme B^+^ and CD107a^+^ CD8^+^ T cells, indicating potent CTL responses that were capable of directly inducing tumor cell apoptosis (Fig. 8N–Q and S10A). mHER2_aHPF nanoparticle vaccines also potently inhibited the expansion of Gr-1^+^CD11b^+^ MDSCs and CD25^+^/Foxp3^+^ Tregs within spleens and tumors, regardless of their combination with ICB therapies (Fig. 8R–U). Moreover, mHER2_aHPF nanoparticle vaccines elicited higher titers of mHER2-specific IgG antibodies compared to the other groups, demonstrating enhanced humoral immune responses (Fig. 8V and S10B). Collectively, our above results demonstrated that therapeutic vaccination with mHER2_aHPF nanoparticle vaccines synergized with ICB therapies to potently suppress tumor progression, which was achieved by activating both cellular and humoral immune responses and inhibiting the expansion of immunosuppressive cell populations.

### 3.8 In vivo therapeutic efficacy of human HER2 nanoparticle vaccines

To investigate whether HER2 nanoparticle vaccines exhibited therapeutic efficacy against human HER2-positive tumors *in vivo*, we established human HER2 tumor models by inoculating female BALB/c mice with hHER2-expressing 4T1 cells. We first compared hHER2_aHPF vaccination head-to-head with trastuzumab, a clinically approved anti-hHER2 monoclonal antibody (mAb) that targets the HER2 extracellular domain. Female BALB/c mice were subcutaneously inoculated with 4T1-hHER2 cells on Day 0. Beginning on Day 1, the adjuvant group received CpG-FNP adjuvant only. The vaccine group received three subcutaneous doses of hHER2_aHPF on Days 1, 7, and 14, while the trastuzumab group received three intraperitoneal injections at 250 μg per mouse (approximately 10 mg/kg) on Days 2, 8, and 15 (Fig. S11A). Tumor volume monitoring showed that both trastuzumab and hHER2_aHPF significantly suppressed tumor growth relative to the adjuvant control (Fig. S11B). Notably, hHER2_aHPF vaccination achieved more pronounced tumor inhibition than trastuzumab, with substantially smaller mean tumor volumes by Day 20.

**Fig. 9:**
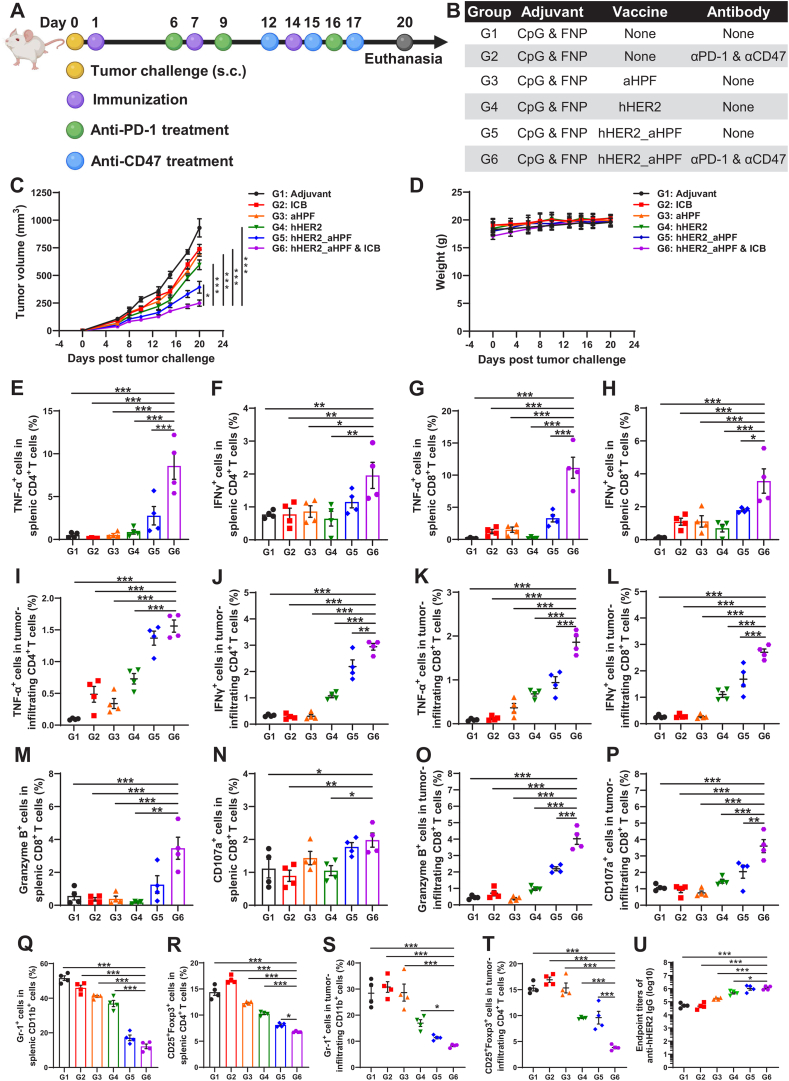
**Therapeutic hHER2 nanoparticle vaccination synergizes with ICB therapies to enhance tumor suppression.** (A) Schematic of therapeutic vaccination combined with ICB therapies (*n* = 4 per group). Female BALB/c mice were subjected to subcutaneous (s.c.) tumor challenge using 4T1-hHER2-Luc cells. Vaccines were administered on Day 1, 7, and 14, while αPD-1 and αCD47 ICB therapies were alternately administered on Day 6, 9, 12, 15, 16, and 17. All mice were euthanized on Day 20. (B) Study groups of vaccination and ICB therapies. G1: adjuvant. G2: αPD-1 and αCD47. G3: aHPF. G4: hHER2. G5: hHER2_aHPF. G6: hHER2_aHPF combined with αPD-1 and αCD47. (C) Average tumor growth curves in different treatment groups. (D) Body weight of mice in different treatment groups from Day 0 to Day 20 after tumor challenge. (E-H) Antigen-specific T cells were evaluated via stimulating lymphocytes with human HER2 peptides. Frequencies of TNF-α^+^CD4^+^ (E), IFNγ^+^CD4^+^ (F), TNF-α^+^CD8^+^ (G), and IFNγ^+^CD8^+^ T cells (H) were quantified by FCM. (I-L) Tumor-infiltrating lymphocytes were stimulated by human HER2 peptides, followed by ICCS and FCM to quantify frequencies of TNF-α^+^CD4^+^ (I), IFNγ^+^CD4^+^ (J), TNF-α^+^CD8^+^ (K), and IFNγ^+^CD8^+^ T cells (L). (M − P) Functional profiling of cytotoxic CD8^+^ T cells in spleens and tumors. Percentages of splenic Granzyme B^+^CD8^+^ T cells (M) and CD107a^+^CD8^+^ T cells (N), as well as tumor-infiltrating Granzyme B^+^CD8^+^ T cells (O) and CD107a^+^CD8^+^ T cells (P) were quantified by FCM. (Q) Percentages of MDSCs in spleens, defined as proportions of Gr-1^+^CD11b^+^ cells within total CD11b^+^ cells. (R) Percentages of Tregs in spleens, analyzed as proportions of CD25^+^Foxp3^+^ cells among total splenic CD4^+^ T cells. (S-T) Percentages of MDSCs (S) and Tregs (T) in tumors were quantified as in (Q-R). (U) ELISA analysis of hHER2-specific IgG in sera of each mouse. Data in (C-U) were presented as mean ± SEM. *P* values were calculated by two-way ANOVA (C) or one-way ANOVA (E-U) with Dunnett's multiple comparisons tests. *P* values in (C) were derived from comparisons conducted on Day 20. *P < 0.05, **P < 0.01, ***P < 0.001.

We next evaluated the therapeutic potential of hHER2_aHPF in combination with ICB therapies. The experimental schedule for tumor challenge, immunization, and ICB therapies was carried out as in Fig. 8A. Briefly, three doses of vaccination were administered to mice on Day 1, 7, and 14 after tumor challenge (Fig. 9A). The ICB therapies were administered alternately during the vaccination period. All mice were euthanized on Day 20 after the tumor volume in the adjuvant group reached 1000 mm^3^. Vaccination groups were set as in Fig. 8B, except that the HER2 antigen employed was derived from human HER2-ECD (Fig. 9B). The monitoring of tumor volumes revealed that mice in the adjuvant (G1), ICB therapies (G2), aHPF (G3), and hHER2 (G4) groups rapidly developed subcutaneous tumors and demonstrated a continuous increase in tumor volumes, while mice vaccinated with hHER2_aHPF (G5) and those receiving its combination with ICB therapies (G6) significantly inhibited tumor progression (Fig. 9C). The combined treatment exhibited the highest therapeutic efficacy among all groups. Throughout the therapeutic course, all groups exhibited consistent body weight gain without notable fluctuations or abrupt declines, suggesting that the administration of hHER2_aHPF vaccines and ICB therapies did not lead to severe systemic side effects or obvious toxicity at the corresponding doses (Fig. 9D).

To elucidate the anti-tumor immune mechanisms underlying the enhanced therapeutic efficacy of hHER2_aHPF nanoparticle vaccines, the splenocytes, tumor-infiltrating cells, and sera from euthanized mice were harvested for the quantification of immune responses. Similar to the observations in mice treated with mHER2_aHPF vaccination and ICB therapies, the combined treatment of hHER2_aHPF vaccines and ICB therapies induced higher percentages of TNF-α^+^ and IFNγ^+^ CD4^+^ T cells, as well as TNF-α^+^ and IFNγ^+^ CD8^+^ T cells, compared with the other groups (Fig. 9E–H). Moreover, the combined treatment also resulted in elevated levels of these cells in tumor-infiltrating T cells, contributing to a pro-immunogenic tumor microenvironment (Fig. 9I–L). Particularly, the combination therapies markedly boosted the cytotoxic capacity of CD8^+^ T cells, as evidenced by the highest frequencies of Granzyme B^+^ and CD107a^+^ CD8^+^ T cells in spleen and tumor tissues among all groups, demonstrating enhanced polyfunctional T-cell responses (Fig. 9M − P). The assessment of immunosuppressive cell populations showed that the combined treatment substantially reduced the frequencies of MDSCs and Tregs in both spleens and tumors, consequently alleviating the immunosuppressive tumor microenvironment (Fig. 9Q–T). The ELISA quantifications revealed that the hHER2_aHPF nanoparticle vaccines effectively elicited higher titers of hHER2-specific IgG antibodies in comparison to the other groups, indicating potent humoral immune responses (Fig. 9U and S11C). Of note, all groups exhibited substantially high titers of hHER2-specific antibodies, primarily originating from the immune responses triggered by the human HER2-expressing tumors in mice. To rule out the possibility that hHER2_aHPF vaccination selected for antigen loss variants, tumor tissues were harvested on Day 37 from a separate cohort of mice immunized prophylactically with hHER2_aHPF or adjuvant, followed by hHER2-expressing tumor challenge. Flow cytometric analysis showed that the percentage of hHER2-positive cells in the hHER2_aHPF group remained comparable to that in the Adjuvant control and the 4T1-hHER2 cell line, indicating no detectable HER2 loss (Fig. S11D and S11E). Taken together, our above results demonstrated that the combined treatment of hHER2_aHPF nanoparticle vaccines and ICB therapies exerted a significant synergistic anti-tumor efficacy in human HER2-targeted tumor models *in vivo*. This combination not only effectively activated antigen-specific cellular and humoral immune responses but also potently inhibited the expansion of immunosuppressive cell populations.

## 4 Discussion

The development of effective cancer vaccines requires overcoming two fundamental obstacles, including the weak immunogenicity of tumor-associated antigens and the profound immunosuppression within the tumor microenvironment (TME). In this study, we have addressed these challenges by engineering a multitargeting ferritin-based nanoparticle vaccine, namely HER2_aHPF, which integrates multivalent antigen display, active dendritic cell (DC) targeting, and synergistic adjuvantation into a unified “immune-organizing” platform. Our findings demonstrate that this rationally designed framework not only elicits robust anti-tumor immune responses but also reshapes the suppressive TME, enabling synergy with dual immune checkpoint blockade (ICB) therapies.

The foundation of the efficacy of HER2_aHPF lies in its structural attributes. Unlike synthetic polymers or liposomes, the *Helicobacter pylori* ferritin (HPF) scaffold offers genetic programmability and atomic-level precision. Our results confirm that the insertion of the AE37 T-cell epitope and the conjugation of the HER2 extracellular domain (ECD) via the SpyTag/SpyCatcher system do not compromise the self-assembly of the 24-meric cage. The resulting 23-nm nanoparticle falls within the optimal size range for rapid lymphatic drainage and uptake by lymph node-resident DCs or follicular dendritic cells (FDCs). This size-dependent lymphatic tropism is critical for vaccine efficacy. Our biodistribution analysis demonstrated that HER2_aHPF exhibited sustained retention in draining lymph nodes at 24 h post-injection compared with monomeric HER2. This prolonged lymph node residence, which is consistent with the passive drainage and subcapsular sinus trapping of 10 to 50 nm particles reported for other molecular vaccines, effectively converts a transient antigen exposure into a sustained stimulatory signal for DCs [71]. Supporting this interpretation, the enhanced DC maturation observed in nanoparticle-vaccinated mice was accompanied by elevated downstream T-cell and antibody responses, indicating that the structural properties of the ferritin cage, specifically its size and multivalency, directly contribute to the immunological potency of HER2_aHPF by prolonging antigen availability in the lymphoid compartment. Moreover, the HER2_aHPF maintains its structural integrity and monodispersity even after repeated freeze-thaw cycles and prolonged storage, addressing a significant bottleneck in the cold-chain transportation and clinical translation of protein-based biologics.

From a manufacturing standpoint, the HER2_aHPF nanoparticle vaccine platform is well suited for scalable production. The HPF scaffold is produced in *E. coli*, while the HER2-ECD antigen is expressed in a mammalian cell system. Both expression hosts are compatible with established GMP manufacturing workflows. Conjugation of the two components via the SpyTag003-SpyCatcher003 system proceeds spontaneously under mild aqueous conditions and does not require chemical cross-linking reagents, thereby simplifying downstream processing. Across the independent batches of aHPF, mHER2_aHPF, and hHER2_aHPF prepared for this study, we observed consistent SEC elution profiles, DLS size distributions, negative staining morphologies, and endotoxin levels. These data support the batch-to-batch reproducibility of the nanoparticle vaccine. Further process development, including clearance of residual host cell proteins and DNA, control of conjugation stoichiometry, and establishment of an *in vitro* potency assay, will be required to advance this platform toward GMP-compliant manufacturing.

A crucial design innovation in HER2_aHPF is the synchronization of B-cell and T-cell activation through the simultaneous presentation of the HER2-ECD and AE37 peptide. HER2-ECD encompasses conformational B-cell epitopes targeted by clinically approved monoclonal antibodies (mAbs) such as trastuzumab and pertuzumab, as well as the E75 CD8^+^ T-cell epitopes [20,24]. Meanwhile, the AE37 CD4^+^ T-cell epitope derived from the HER2 intracellular domain is genetically fused to the ferritin core [22]. Sequence alignment and structural analysis confirm that mHER2 and hHER2 share 87.66% amino acid identity, with the E75 and AE37 epitopes completely conserved between the two species. This high degree of homology is reflected in the comparable magnitudes of humoral and cellular immunity elicited by mHER2_aHPF and hHER2_aHPF in BALB/c mice. The slightly stronger responses observed with hHER2_aHPF in some assays are consistent with the xenogeneic nature of the human antigen, which presents additional heterologous epitopes beyond the conserved domains. These findings support the use of murine HER2 as a valid surrogate for evaluating vaccine-induced immunity while also confirming that the hHER2 construct elicits potent immunity against the clinically relevant human antigen. By displaying the HER2-ECD in a multivalent and virus-like arrangement, HER2_aHPF facilitates efficient B-cell receptor (BCR) cross-linking and antigen retention by FDCs in lymph nodes, thereby augmenting germinal center (GC) reactions and the production of high-titer and high-affinity HER2-specific antibodies. These antibodies are essential for mediating antibody-dependent cellular cytotoxicity (ADCC) and antibody-dependent cellular phagocytosis (ADCP), as demonstrated by the enhanced macrophage phagocytosis observed *in vitro*. Concurrently, the incorporation of the AE37 epitope and the HER2-ECD-derived E75 epitope facilitates extensive priming of CD4^+^ T-helper and CD8^+^ cytotoxic T lymphocytes. Our data reveal that HER2_aHPF elicits robust polyfunctional T-cell responses, characterized by increased frequencies of TNF-α^+^ and IFNγ^+^ T lymphocytes in spleens and tumors, as well as elevated Granzyme B^+^ and CD107a^+^ CD8^+^ T cells, indicating strong cytolytic potential and degranulation capacity. The vaccine also stimulates the expansion of effector-memory T (T_EM_) cells, suggesting that it can generate durable immunological memory rather than transient effector responses.

The enhancement of immunogenicity also relies on efficient antigen presentation and adjuvantation. In addition to the ferritin scaffold, we employ a three-pronged strategy to maximize DC activation, including WH peptide targeting, CpG stimulation, and fullerenol modulation. The WH peptide targets Clec9a, a receptor highly expressed on the CD8α^+^ DC and CD103^+^ DC subsets specialized in cross-presentation [50]. By directing vaccines towards these DC populations, HER2_aHPF demonstrates enhanced internalization by DC2.4 cells and promotes DC maturation, as evidenced by increased CD80/CD86 expression and elevated secretion of IL-6 and TNF-α. CpG oligodeoxynucleotides (CpG ODNs) are well-established TLR9 agonists that promote the production of type I interferon (IFN-I) and proinflammatory cytokines by antigen-presenting cells (APCs), thereby enhancing T-cell priming and Th1-biased responses [51]. Our previous work and current findings suggest that fullerenol creates a “pro-immunogenic” environment, likely by inducing cellular stress that facilitates the exposure of the “eat me” signal calreticulin on tumor cells [52,56]. In this study, we demonstrate that fullerenol also functions as a potent immunoadjuvant for HER2_aHPF, significantly enhancing both cellular and humoral immune responses. When CpG and fullerenol are co-formulated with WH peptide-tagged HER2_aHPF, the resulting dual-adjuvanted nanoparticle vaccines induce robust polyfunctional T-cell responses and elevated HER2-specific antibody titers. Therefore, this synergistic targeting and adjuvant strategy establishes a highly coordinated immune activation loop that connects antigen delivery, innate sensing, and adaptive effector generation.

In addition to the magnitude of anti-tumor immune responses, the quality of the tumor microenvironment (TME) is a critical determinant of vaccine efficacy. The TME of established tumors is generally dominated by immunosuppressive cell populations, including myeloid-derived suppressor cells (MDSCs) and regulatory T cells (Tregs), which physically and chemically impede the function of cytotoxic T lymphocytes (CTLs) [63,68]. Our study demonstrates that HER2_aHPF vaccination significantly reshapes the systemic and intratumoral immune landscapes. In both prophylactic and therapeutic tumor models, HER2_aHPF reduces the accumulation of MDSCs and Tregs in spleens and tumors. This simultaneous reduction in MDSCs and Tregs alleviates the key suppressive pathways that usually dampen vaccine-induced effector responses, thereby increasing the numbers of tumor-infiltrating CD8^+^ T cells. These reductions likely result from the strong Th1-biased IFN-γ cytokine profiles, which counteract the differentiation of MDSCs and Tregs. Additionally, the effective elimination of tumor cells by vaccine-induced CTLs reduces the source of tumor-derived TGF-β and GM-CSF that recruit these suppressor cells. The remodeling of the TME renders the tumor more vulnerable to effector cell attack, consequently inhibiting tumor progression.

The 4T1 syngeneic model used in this study recapitulates several clinically relevant features of human HER2-positive breast cancer that support the translational interpretation of our findings. The 4T1 line is spontaneously metastatic, poorly immunogenic, and characterized by robust intratumoral expansion of MDSCs and Tregs, closely mirroring the immunosuppressive tumor microenvironment observed in patients with advanced HER2-positive disease. The upregulation of PD-L1 and CD47 on 4T1 cells further parallels the immune evasion pathways documented in clinical specimens, providing a mechanistically relevant setting in which to evaluate the synergy between active vaccination and dual checkpoint blockade. By engineering 4T1 cells to stably express human HER2 in parallel with the endogenous murine antigen, we were able to assess vaccine efficacy against both murine and human HER2 in fully immunocompetent hosts. This dual-antigen approach preserves the intact immune compartments required for evaluating vaccine-induced adaptive immunity while maintaining a direct translational bridge to the human target antigen.

Despite the potent immune responses induced by vaccines, tumor eradication in established models remains challenging due to adaptive immune resistance, which is characterized by the upregulation of checkpoint ligands such as PD-L1 and CD47 on tumor cells. PD-L1 interacts with PD-1 on T cells, leading to T-cell exhaustion (T_EX_) and restricting effector function [64]. Meanwhile, CD47 serves as a “don't eat me” signal and binds to SIRPα on macrophages to suppress their phagocytosis [65]. The immune checkpoint blockade (ICB) therapies with mAbs targeting both PD-1 and CD47 have demonstrated remarkable efficacy in restoring anti-tumor immunity [66,67]. Therefore, we propose to combine active vaccination with ICB therapies to form a self-reinforcing therapeutic loop. Our results confirm that the combination of HER2_aHPF with dual PD-1/CD47 blockade in both mouse and human HER2-positive tumor models leads to the highest rate of tumor regression and lung metastasis inhibition. In both prophylactic and therapeutic models, the combined treatment consistently demonstrates the most favorable balance of polyfunctional T cells and durable immunological memory, decreases MDSCs and Tregs, and maintains HER2-specific antibody titers. The combined efficacy operates as a closed-loop system. Vaccination increases tumor-specific T cells and antibodies. PD-1 blockade restores and preserves T-cell function in chronically inflamed tumors, and CD47 blockade boosts macrophage-mediated clearance and antigen recycling, which in turn sustains DC priming and T-cell expansion. These results emphasize the robustness of the synergistic interaction between nanoparticle vaccines and ICB therapies.

In addition to vaccination combined with ICB, the compatibility of HER2_aHPF with existing HER2-targeted therapies warrants consideration. With respect to mAbs, our head-to-head comparison demonstrated that hHER2_aHPF vaccination achieved tumor control comparable to that of trastuzumab monotherapy. However, trastuzumab and pertuzumab provide only passive antitumor activity without establishing immunological memory, whereas HER2_aHPF induces durable effector-memory T-cell responses and high-titer HER2-specific antibodies. Because HER2_aHPF displays the full HER2-ECD, vaccine-induced polyclonal antibodies may recognize epitopes that overlap with those targeted by therapeutic mAbs. In contrast to short peptide vaccines that target a single T-cell epitope and do not compete with therapeutic antibodies for cell-surface HER2, the full-length ECD display strategy introduces a potential for epitope competition that warrants consideration in clinical trial design. Sequential administration, in which mAbs are given first to achieve initial tumor burden reduction and are allowed to clear before vaccination, would circumvent this concern and represents a rational clinical strategy. Antibody-drug conjugates (ADCs) such as trastuzumab deruxtecan (T-DXd) offer additional opportunities for synergy. T-DXd carries a membrane-permeable topoisomerase I inhibitor payload that mediates bystander killing of neighboring HER2-low or HER2-negative tumor cells, a property directly relevant to the challenge of intratumoral HER2 heterogeneity [72]. Furthermore, ADC-mediated tumor cell killing can induce immunogenic cell death, which is accompanied by the release of tumor-associated antigens and damage-associated molecular patterns that may enhance DC maturation and amplify the adaptive immune response primed by subsequent vaccination [73]. Small-molecule HER2 tyrosine kinase inhibitors (TKIs) such as lapatinib and tucatinib target the intracellular kinase domain of HER2 and do not compete with vaccine-induced antibodies for extracellular epitopes, suggesting that TKI therapy could be administered concurrently with HER2_aHPF vaccination without the risk of epitope interference [74]. These considerations indicate that HER2_aHPF vaccination can be rationally integrated into the existing HER2-targeted treatment paradigm, with the optimal strategy likely involving sequential administration for mAbs and ADCs, and concurrent administration for TKIs.

The clinical translation of the three-dose immunization schedule employed in this study also deserves consideration. The 0-, 7-, and 14-day prime-boost regimen used here was designed to maximize immunogenicity within the relatively short timeframe of murine studies. In humans, protein subunit vaccines are typically administered at longer intervals, with standard schedules such as 0, 1, and 6 months for hepatitis B virus vaccine and 0, 2, and 6 months for human papillomavirus vaccine, reflecting the kinetics of germinal center reactions and memory B-cell maturation in primates. Ferritin-based nanoparticle vaccines are currently under active clinical evaluation, and preliminary data suggest that their highly multivalent antigen display may support immunogenic dosing schedules that are compatible with clinical practice [75]. In the present study, the three-dose regimen generated robust effector-memory T-cell responses and high-titer HER2-specific antibodies with a favorable safety profile, including normal cardiac histology and serum biochemistry after repeated dosing. Nevertheless, several challenges must be addressed to translate this regimen to clinical practice. Multiple clinic visits for repeated subcutaneous injections may reduce patient adherence and increase healthcare costs, and repeated injections at the same site carry a risk of local reactogenicity. Future development efforts may benefit from sustained-release formulations, such as injectable hydrogel depots or polymeric microsphere systems, which could reduce the number of injections required while maintaining or even enhancing immunogenicity through prolonged antigen exposure. Additionally, given that HER2 is expressed at basal levels in cardiomyocytes and that trastuzumab is associated with a well-documented risk of cardiotoxicity, the long-term cardiac safety of repeated HER2_aHPF booster dosing should be evaluated in extended-duration animal models before clinical implementation.

Collectively, our study has demonstrated the high efficacy of nanoparticle-based vaccines against HER2-positive breast cancer. However, several limitations should be further addressed to facilitate their clinical translation. Although the 4T1 models are aggressive, they do not fully recapitulate the heterogeneity of human breast cancer. In clinical HER2-positive breast cancer, intratumoral heterogeneity in HER2 expression is well documented, with HER2-high, HER2-low, and HER2-negative subclones frequently coexisting within the same lesion. Because HER2_aHPF-induced ADCP and CTL responses are fundamentally HER2-directed, HER2-negative or HER2-low tumor subpopulations may evade vaccine-mediated killing and serve as a reservoir for disease progression. This inherent limitation is partially mitigated by the combination of vaccination with dual PD-1/CD47 immune checkpoint blockade, which can broaden the antitumor T-cell repertoire beyond HER2-restricted specificities through the relief of T-cell exhaustion and the promotion of epitope spreading. The release of tumor-associated antigens and damage-associated molecular patterns from HER2-positive cells killed by vaccine-induced effectors may further facilitate the cross-priming of T cells against non-HER2 neoantigens, thereby extending immune pressure to antigenically distinct subclones. The homogeneous HER2 overexpression in the 4T1-HER2 model used in this study was therefore a deliberate experimental choice, as it enabled unambiguous assessment of vaccine immunogenicity and antitumor efficacy in a defined antigenic context without the confounding variable of preexisting antigen heterogeneity. Future studies employing mixed HER2-positive and HER2-negative tumor cells, or genetically engineered models with heterogeneous HER2 expression, will be necessary to directly evaluate the capacity of HER2_aHPF-based vaccination, alone and in combination with ICB, to control tumors with heterogeneous target antigen expression. The use of humanized mouse models reconstituted with human immune cells and challenged with patient-derived HER2-positive tumors would provide a more clinically relevant assessment of vaccine efficacy and safety, and would represent an important direction for future preclinical development.

Meanwhile, longitudinal studies are essential to define the durability of protection, the rate of epitope spreading, and the potential induction of antigen loss variants following HER2_aHPF vaccination. Although flow cytometric analysis of tumor cells from vaccinated mice showed no evidence of HER2 downregulation, IHC analysis and longer-term observation would further strengthen this assessment. Furthermore, the mechanistic characterization of the CpG-FNP dual-adjuvant system was based on transcriptional profiling by RT-qPCR. Genetic ablation using TLR knockout models and protein-level validation by western blotting were beyond the scope of the present study, and dedicated investigations employing these approaches will be needed to fully elucidate the signaling pathways underlying FNP adjuvanticity. Additionally, the ferritin scaffold provides a practical framework for incorporating multiple tumor antigens or neoantigen cocktails, which could be particularly significant for tackling intratumoral heterogeneity and preventing immune escape. Finally, rational combinations of HER2_aHPF with other immunomodulators, such as costimulatory agonists, STING agonists, or localized delivery approaches, could further boost efficacy and broaden the spectrum of responsive tumor types.

## 5 Conclusion

In summary, this study presents a versatile ferritin-based HER2_aHPF nanoparticle vaccine that co-displays the HER2-ECD and the AE37 T-cell epitope on a structurally well-defined and exceptionally stable protein nanoparticle. By integrating WH peptide-mediated DC targeting with a CpG-fullerenol dual-adjuvant system, this vaccine exhibits enhanced lymphatic drainage, elicits potent HER2-specific humoral and cellular immune responses, and maintains a favorable safety profile. In mouse models of HER2-positive breast cancer, HER2_aHPF exhibits robust prophylactic and therapeutic efficacy in impeding tumor progression. Moreover, it synergizes with anti-PD-1 and anti-CD47 dual ICB therapies to achieve superior suppression of primary tumor growth and lung metastasis. These findings indicate that the rationally engineered ferritin nanoparticle vaccines can orchestrate coordinated innate and adaptive immune responses and function as effective adjuncts for ICB therapies, thereby supporting the translational potential of HER2_aHPF and related constructs for the treatment of HER2-positive malignancies.

## CRediT authorship contribution statement

**Sen Liu:** Conceptualization, Data curation, Formal analysis, Investigation, Methodology, Visualization, Writing – original draft. **Yiqiang Zhu:** Data curation, Formal analysis, Investigation, Methodology, Software. **Tao Chen:** Formal analysis, Investigation, Methodology, Validation, Visualization. **Xiaoqing Liu:** Investigation, Methodology, Validation. **Jintao Lai:** Investigation, Resources, Software. **Meilin Hu:** Data curation, Investigation. **Yaoming Liu:** Resources, Validation. **Haiyue Rao:** Resources. **Bin Zhang:** Visualization. **Shiqi Xiao:** Investigation. **Haojie Peng:** Investigation. **Taizhen Liang:** Software. **Lixiang Xie:** Software. **Guochang Qiu:** Visualization. **Shan Li:** Conceptualization, Formal analysis, Methodology, Resources, Supervision, Visualization, Writing – original draft, Writing – review & editing. **Xiancai Ma:** Conceptualization, Formal analysis, Funding acquisition, Methodology, Resources, Supervision, Visualization, Writing – original draft, Writing – review & editing.

## Declaration of competing interest

The authors declare that they have no known competing financial interests or personal relationships that could have appeared to influence the work reported in this paper.

## Data Availability

Data will be made available on request.

## Appendix A Supplementary data

Supplementary data related to this article included: Fig. S1. Homology and structural analyses of mouse and human HER2 proteins. Fig. S2. Construction and stability of HER2 nanoparticle vaccines. Fig. S3. Cytotoxicity assessment of nanoparticle vaccines and gating strategies for BMDCs. Fig. S4. Gating strategies used for immune cell quantifications. Fig. S5. The enhanced efficacy of ADCP induced by mHER2_aHPF nanoparticle vaccines. Fig. S6. The enhanced efficacy of ADCP induced by hHER2_aHPF nanoparticle vaccines. Fig. S7. Safety evaluation of HER2 nanoparticle-vaccinated mice. Fig. S8. Gating strategies for MDSCs and CD8^+^ T_EM_ cells. Fig. S9. Gating strategies for Tregs and anti-mHER2 IgG titers. Fig. S10. Gating strategies for degranulating CD8^+^ T cells and anti-mHER2 IgG titers. Fig. S11. Head-to-head comparison between hHER2_aHPF and HER2-targeted therapies. Table S1. Primers used for RT-qPCR.Multimedia component 1
