# Supplementary material for: A multitargeting nanoparticle-based vaccine for enhanced anticancer immunotherapy in HER2-Positive breast cancer
Source: Mater Today Bio. 2026 Aug 3;40:103524. doi: 10.1016/j.mtbio.2026.103524 (PMC13473953; doi:10.1016/j.mtbio.2026.103524)
Supplement: Supplementary file 1 [file mmc1.docx]

Supporting Information

A Multitargeting Nanoparticle-Based Vaccine for Enhanced Anticancer Immunotherapy in HER2-Positive Breast Cancer

Sen Liu ^a,b,c,1^, Yiqiang Zhu ^b,c,1^, Tao Chen ^b,c^, Xiaoqing Liu ^b,c^, Jintao Lai ^c^, Meilin Hu ^b,c^, Yaoming Liu ^c^, Haiyue Rao ^c^, Bin Zhang ^c^, Shiqi Xiao ^c^, Haojie Peng ^c,d^, Taizhen Liang ^c,d^, Lixiang Xie ^c^, Guochang Qiu ^c,d^, Shan Li ^a,^*, and Xiancai Ma ^a,b,c,d,^*

^a^ School of Biology and Biological Engineering, South China University of Technology, Guangzhou 510006, China

^b^ Institute of Human Virology, Zhongshan School of Medicine, Sun Yat-sen University, Guangzhou 510080, China

^c^ Guangzhou National Laboratory, Guangzhou International Bio-Island,

Guangzhou 510005, China

^d^ State Key Laboratory of Respiratory Disease, National Clinical Research Center for Respiratory Disease, Guangzhou Institute of Respiratory Health, The First Affiliated Hospital of Guangzhou Medical University, Guangzhou 510120, China

*** Corresponding authors. E-mail addresses: [lishan@scut.edu.cn](mailto:lishan@scut.edu.cn) (S. Li), [ma_xiancai@gzlab.ac.cn](mailto:ma_xiancai@gzlab.ac.cn) (X. Ma)

^1^ These authors contributed equally to this work.

**Figure S1. Homology and structural analyses of mouse and human HER2 proteins.** (A) Amino acid sequence alignment of mHER2 (NP_001003817.1) and hHER2 (NP_004439.2). The alignment revealed 87.66% identity across the full-length proteins. Highly conserved regions were shown in red. The three highlighted epitopes in blue corresponded to the CD8^+^ T-cell epitope E75 (KIFGSLAFL), the CD4^+^ T-helper epitope AE36 (GVGSPYVSRLLGICL), and the GP2 peptide (mouse: IIATVVGVL; human: IISAVVGIL). E75 and AE36 were identical between mouse and human HER2, whereas GP2 differed by several residues. (B) AlphaFold 3 (AF3)-predicted three-dimensional (3D) structures of mHER2 (purple) and hHER2 (blue). Magnified views showed the spatial conformation of the E75, GP2, and AE36 epitopes within each protein. E75 was fully solvent-exposed on the extracellular domain (ECD) surface in both structures. AE36, which was naturally located in the HER2 intracellular domain, was not surface-accessible in the full-length protein but was genetically fused to the ferritin nanoparticle core in the vaccine construct. GP2, which diverged between mouse and human HER2, resided in the transmembrane region and was not included in the vaccine design.

**Figure S2. Construction and stability of HER2 nanoparticle vaccines.** (A) SDS-PAGE analysis of SpyTag003-SpyCatcher003 conjugation between 1 nmol aHPF and increasing molar amounts (0, 1, 2, 3, and 4 nmol) of mHER2 or hHER2. Reactions were incubated at 4°C for 24 h. (B-C) Densitometric quantification of conjugation efficiency for mHER2_aHPF (B) and hHER2_aHPF (C) as a function of the HER2-to-aHPF molar ratio. (D-G) Time course of the conjugation reaction at a fixed ratio of 3 nmol mHER2 (D) or hHER2 (F) to 1 nmol aHPF. Quantitative kinetic analysis of mHER2_aHPF (E) and hHER2_aHPF (G) conjugation efficiency over 0-180 min. (H-K) SDS-PAGE analysis of mHER2_aHPF (H) and hHER2_aHPF (J) stored under the indicated conditions: freshly prepared, -80°C for 2 months, -20°C for 1 month, and 4°C for 1 week. Densitometric quantification of the proportions of intact and degraded proteins was shown for mHER2_aHPF (I) and hHER2_aHPF (K). (L) Endotoxin levels in aHPF, mHER2_aHPF, and hHER2_aHPF measured across three independent batches per preparation using a chromogenic Tachypleus amebocyte lysate (TAL) assay. (M-N) ELISA titration curves (OD450 vs. serum dilution) for anti-mHER2 IgG (M) and anti-hHER2 IgG (N) in sera from mice (*n* = 4 per group) immunized with vaccines subjected to 0, 1, 3, 5, 7, and 9 freeze-thaw cycles. Endpoint titers were determined by non-linear regression analysis of the dilution curves. Data in (L-N) were presented as mean ± SEM.

**Figure S3. Cytotoxicity assessment of nanoparticle vaccines and gating strategies for BMDCs.** (A-D) DC2.4 cells (A-B) and RAW264.7 cells (C-D) were incubated with 200 μg/mL of mHER2_aHPF or hHER2_aHPF for 24 h (A, C) or 48 h (B, D), followed by staining with an Annexin V-FITC/PI kit and analyzed by flow cytometry. Representative dot plots were shown. The percentages of live (Annexin V^-^/PI^-^), dead (Annexin V^-^/PI^+^), early apoptotic (Annexin V^+^/PI^-^), and late apoptotic (Annexin V^+^/PI^+^) cells were quantified. Treatment with PBS served as a negative control. (E-F) Red blood cells isolated from mice were incubated with mHER2_aHPF (E) or hHER2_aHPF (F) at concentrations of 200, 500, 1,000, 2,500, 5,000, and 10,000 μg/mL at 37°C for 1 h. PBS-treated and ddH_2_O-treated red blood cells served as the negative and positive controls, respectively. After incubation, the absorbance values of the clarified supernatants were measured at 540 nm. The hemolysis ratios were expressed as percentages relative to the positive control (set to 100%). (G) Gating strategies for mature BMDCs, which were defined as CD11c^+^CD80^+^CD86^+^ cells. Data in (E-F) were presented as mean ± SEM.

**Figure S4. Gating strategies used for immune cell quantifications.** (A) Representative gating hierarchy for antigen-specific T-cell subsets. Lymphocytes in spleen or tumor tissues were stimulated with mouse or human HER2 peptide pools, followed by intracellular cytokine staining (ICCS) assays to probe cytokine-expressing cells. The TNF-α^+^, IFNγ^+^, and Granzyme B^+^ cells were analyzed by FCM for CD3^+^CD8^+^ T cells, while TNF-α^+^ and IFNγ^+^ cells were analyzed for CD3^+^CD4^+^ T cells. (B) ELISA titration curves (OD450 vs. serum dilution) for anti-mHER2 IgG in mice immunized with 10 μg mHER2_aHPF adjuvanted with 0, 40, 60, or 80 μg FNP. Data in (B) were presented as mean ± SEM.

**Figure S5. The enhanced efficacy of ADCP induced by mHER2_aHPF nanoparticle vaccines.** (A) Twenty-four hours after subcutaneous injection of Cy7-labeled mHER2 and mHER2_aHPF (0.4 nmol per mouse) in the cervicodorsal region of female BALB/c mice, the lymph node, spleen, liver, kidney, lung, and heart were excised and imaged *ex vivo*. The relative distribution of fluorescence signal among organs was expressed as a percentage of the total radiance summed across all six organs, with the lymph node and spleen distributions highlighted to emphasize lymphatic targeting. (B) Gating strategies for DC populations in lymph nodes. CD45^+^CD11c^+^ DCs from lymph nodes of vaccinated mice were further gated to select CD80^+^CD86^+^ cells, which were defined as mature DCs. (C) ELISA titration curves (OD450) against serial serum dilutions for anti-mHER2 IgG. Mice were immunized with Adjuvant (G1), aHPF (G2), mHER2 (G3), or mHER2_aHPF (G4). (D) Gating strategies for tumor cell-phagocytosed macrophages. The *in vitro* antibody-dependent cellular phagocytosis (ADCP) assay was conducted to quantify the efficacy of phagocytosis mediated by nanoparticle vaccines. 4T1-mHER2-Luc cells were incubated with 5-fold diluted sera from each mouse for 12 h at 37°C, followed by labeling with CellTrace Violet (CTV). CTV-labeled tumor cells were co-cultured with RAW264.7 cells at an effector:target ratio of 1:1 for 2 h at 37°C, followed by FCM assay to quantify the percentages of CTV^+^F4/80^+^ macrophages. (E-F) 4T1-mHER2-Luc cells were pretreated with sera of vaccinated mice (Adjuvant, aHPF, mHER2, and mHER2_aHPF), followed by staining with CTV and co-culturing with RAW264.7 cells. Macrophages that phagocytosed tumor cells were analyzed by FCM (E). The percentages of tumor cell-phagocytosed macrophages were presented as the proportions of CTV^+^F4/80^+^ cells (F). (G-H) RAW264.7 macrophages were pre-incubated with 2.5 μg/mL anti-mouse CD16/CD32 antibody for 10 min at 4°C prior to the phagocytosis assay. Representative flow cytometry plots (G) and quantification (H) of CTV^+^F4/80^+^ macrophages after Fc receptor blockade were shown. Data in (C), (F), and (H) were presented as mean ± SEM. *P* values were calculated by one-way ANOVA with Tukey's multiple comparisons tests. ** *P* < 0.01, *** *P* < 0.001.

**Figure S6. The enhanced efficacy of ADCP induced by hHER2_aHPF nanoparticle vaccines.** (A) Cy7-labeled hHER2 and hHER2_aHPF (0.4 nmol each) were administered subcutaneously in the cervicodorsal region of female BALB/c mice. At 24 h, the heart, lung, liver, kidney, spleen, and lymph node were collected for *ex vivo* imaging. The fluorescence signal in each organ was quantified as a percentage of the total radiance across all six organs, with lymphatic distribution emphasized in the lymph node and spleen. (B) Anti-hHER2 IgG endpoint titers measured by ELISA across serial serum dilutions in mice receiving Adjuvant (G1), aHPF (G2), hHER2 (G3), or hHER2_aHPF (G4). (C-D) The 4T1-hHER2-Luc tumor cells were incubated with sera from vaccinated mice (Adjuvant, aHPF, hHER2, and hHER2_aHPF), followed by staining with CTV and co-culturing with RAW264.7 cells. The percentages of CTV^+^F4/80^+^ cells were quantified by FCM (C) and presented as the proportions of tumor cell-phagocytosed macrophages (D). (E-F) To assess the contribution of FcγR-mediated recognition, RAW264.7 macrophages were treated with 2.5 μg/mL anti-mouse CD16/CD32 antibody at 4°C for 10 min before the co-culture step. Flow cytometry profiles (E) and the corresponding percentages of CTV^+^F4/80^+^ macrophages (F) under Fc receptor blockade conditions were presented. Data in (B), (D), and (F) were presented as mean ± SEM. *P* values were calculated by one-way ANOVA with Tukey's multiple comparisons tests. ** *P* < 0.01, *** *P* < 0.001.

**Figure S7. Safety evaluation of HER2 nanoparticle-vaccinated mice.** (A) Hematoxylin and eosin (H&E) staining of the myocardium, liver, spleen, lung, and kidney from mice in the Mock, Adjuvant, mHER2_aHPF, and hHER2_aHPF groups. Scale bar, 50 μm. (B-E) Hematological parameters including red blood cell (RBC) (B), platelet (PLT) (C), hemoglobin (HGB) (D), and hematocrit (HCT) (E). Dashed lines indicated the normal reference ranges. (F-M) Serum biochemical indicators of liver and kidney function, including alanine aminotransferase (ALT) (F), aspartate aminotransferase (AST) (G), alkaline phosphatase (ALP) (H), total bilirubin (TBIL) (I), γ-glutamyl transferase (γ-GT) (J), total bile acids (TBA) (K), urea (UREA) (L), and creatinine (CREA) (M). Dashed lines indicated the normal reference ranges. (N-O) Serum levels of the pro-inflammatory cytokines IL-6 (N) and TNF-α (O) in mice from the indicated groups. Data in (B-O) were expressed as mean ± SEM. *P* values in (N-O) were calculated by one-way ANOVA with Dunnett's multiple comparisons tests. *** *P* < 0.001.

**Figure S8. Gating strategies for MDSCs and CD8^+^ T_EM_ cells.** (A) Gating strategies for MDSCs. The CD45^+^/CD11b^+^/Gr-1^+^ cells within spleens or tumors were gated and defined as MDSCs. (B) Gating strategies for CD8^+^ T_EM_ cells. CD44^+^/CD62L^-^ populations within CD3^+^/CD8^+^ T cells from spleens were gated and represented as CD8^+^ effector-memory T (T_EM_) cells. (C) ELISA titration curves (OD450) of anti-mHER2 IgG in sera from mice immunized with different formulations: G1 (Adjuvant), G2 (aHPF), G3 (mHER2), and G4 (mHER2_aHPF). (D) In a separate cohort of the mHER2 prophylactic model, immunization was performed on Days 0, 7, and 14 with mHER2_aHPF or adjuvant. Mice were challenged subcutaneously with 4T1-mHER2 cells on Day 15 and euthanized on Day 37. Representative flow cytometry histograms of mHER2 expression in tumor tissues from the Adjuvant and mHER2_aHPF groups and in the 4T1-mHER2 cell line were shown. Unstained cells served as a negative control. (E) Quantification of the percentages of mHER2-positive cells in tumor tissues from the Adjuvant and mHER2_aHPF groups and in the 4T1-mHER2 cell line. Data in (C) and (E) were presented as mean ± SEM.

**Figure S9. Gating strategies for Tregs and anti-mHER2 IgG titers.** (A) Gating strategies for Tregs. CD25^+^/Foxp3^+^ cells were further gated from CD45^+^/CD3^+^/CD4^+^ T cells and defined as regulatory T cells (Tregs). (B) ELISA titration curves (OD450 vs. serum dilution) for anti-mHER2 IgG across all six immunization groups: G1 (Adjuvant), G2 (anti-PD-1 and anti-CD47), G3 (aHPF), G4 (mHER2), G5 (mHER2_aHPF), and G6 (mHER2_aHPF combined with anti-PD-1 and anti-CD47). Data in (B) were presented as mean ± SEM.

**Figure S10. Gating strategies for degranulating CD8^+^ T cells and anti-mHER2 IgG titers.** (A) Gating strategies for degranulating CD8^+^ T cells. CD107a^+^ cells were further gated from CD3^+^CD8^+^ T cells and defined as degranulating CD8^+^ T cells. (B) Anti-mHER2 IgG endpoint titers determined by ELISA in sera from the six treatment groups: Adjuvant (G1), anti-PD-1 and anti-CD47 (G2), aHPF (G3), mHER2 (G4), mHER2_aHPF (G5), and mHER2_aHPF plus anti-PD-1 and anti-CD47 (G6). Data in (B) were presented as mean ± SEM.

**Figure S11. Head-to-head comparison between hHER2_aHPF and HER2-targeted therapies.** (A) Schematic of experimental design. Female BALB/c mice (*n* = 4 per group) were subcutaneously inoculated with 1 × 10^5^ 4T1-hHER2 cells on Day 0. Beginning on Day 1, the adjuvant group received CpG-FNP adjuvant only. The vaccine group received three subcutaneous doses of 1 nmol hHER2_aHPF co-administered with CpG-FNP adjuvant on Days 1, 7, and 14. The trastuzumab group received three intraperitoneal injections of trastuzumab at 250 μg per mouse (approximately 10 mg/kg) on Days 2, 8, and 15. All mice were euthanized on Day 20. (B) Tumor volumes measured at the indicated time points. (C) ELISA titration curves (OD450 vs. serum dilution) for anti-hHER2 IgG across all six immunization groups: G1 (Adjuvant), G2 (anti-PD-1 and anti-CD47), G3 (aHPF), G4 (hHER2), G5 (hHER2_aHPF), and G6 (hHER2_aHPF combined with anti-PD-1 and anti-CD47). (D) Mice were immunized with three doses of hHER2_aHPF or adjuvant on Days 0, 7, and 14, followed by subcutaneous tumor challenge with 4T1-hHER2 cells on Day 15. Tumor tissues were harvested on Day 37. Representative flow cytometry histograms of hHER2 expression in tumor tissues from the Adjuvant and hHER2_aHPF groups and in the 4T1-hHER2 cell line were shown. Unstained cells served as a negative control. (E) Quantification of the percentages of hHER2-positive cells in tumor tissues from the Adjuvant and hHER2_aHPF groups and in the 4T1-hHER2 cell line. Data in (C) and (E) were expressed as mean ± SEM.

**Table S1. Primers used for RT-qPCR.**

| **Gene name** | **Accession** | **Forward primer** | **Reverse primer** |
| --- | --- | --- | --- |
| Mouse *Tlr1* | NM_001276445.1 | AAGGTTGTCTTGACGGAACA | CTGCTCGCCTGAGTTCTTCA |
| Mouse *Tlr2* | NM_011905.3 | GCATCCGAATTGCATCACCG | ACTTTACCCAGCTCGCTCAC |
| Mouse *Tlr3* | NM_001357316.1 | GCGCATATCACAGGCTGAA | TGTCAGGTTCGTGCAGAAGA |
| Mouse *Tlr4* | NM_021297.3 | CTAACTTCCCTCCTGCGACG | TCAAGGGGTTGAAGCTCAGAT |
| Mouse *Tlr5* | NM_016928.4 | GAATCCCGCTTGGGAGAACA | TTGTGATGTCCACCGTCCAG |
| Mouse *Tlr6* | NM_001359180.3 | TCGGAGAAGGAAGTCTTGAGC | TATTAAGGCCAGGGCGCAAA |
| Mouse *Tlr7* | NM_001290755.1 | CAGAGGCCCATGTGATCGT | GCCCTCAGGGATTTCTGTCA |
| Mouse *Tlr8* | NM_001313760.1 | AGCCAGAAAGGGGAAAACATGC | ATTTGGGTGCTGTTGTTTGGC |
| Mouse *Tlr9* | NM_031178.2 | CTCCAACCGTATCCACCACC | GAGAAGTGCAGGGGGCTAAG |
| Mouse *Nlrp3* | NM_001359638.1 | AGGCTGCTATCTGGAGGAACT | TAGACTCCTTGGCGTCCTGA |
| Mouse *Il12b* | NM_001303244.1 | ACGGCAGTGTGCTTGTCTAA | ATGGCGAACCTGGATGGTTT |
| Mouse *Ifnb1* | NM_010510.2 | GCACTGGGTGGAATGAGACT | AGGTACCTTTGCACCCTCCA |
| Mouse *Il1b* | NM_008361.4 | TGCCACCTTTTGACAGTGATG | TTCTTGTGACCCTGAGCGAC |
| Mouse *Il6* | NM_001314054.1 | GCCTTCTTGGGACTGATGCT | TGTGACTCCAGCTTATCTCTTGG |
| Mouse *Gapdh* | NM_001289726.2 | AAGAGGGATGCTGCCCTTAC | TACGGCCAAATCCGTTCACA |
